# Dual-targeted near-infrared photoimmunotherapy for esophageal cancer and cancer-associated fibroblasts in the tumor microenvironment

**DOI:** 10.1038/s41598-022-24313-3

**Published:** 2022-11-23

**Authors:** Hiroaki Sato, Kazuhiro Noma, Toshiaki Ohara, Kento Kawasaki, Masaaki Akai, Teruki Kobayashi, Noriyuki Nishiwaki, Toru Narusaka, Satoshi Komoto, Hajime Kashima, Yuki Katsura, Takuya Kato, Satoru Kikuchi, Hiroshi Tazawa, Shunsuke Kagawa, Yasuhiro Shirakawa, Hisataka Kobayashi, Toshiyoshi Fujiwara

**Affiliations:** 1 Department of Gastroenterological Surgery, Okayama University Graduate School of Medicine, Dentistry and Pharmaceutical Sciences, 2-5-1 Shikata-Cho, Kita-Ku, Okayama, 700-8558 Japan; 2 Department of Pathology & Experimental Medicine, Okayama University Graduate School of Medicine, Dentistry and Pharmaceutical Sciences, Okayama, Japan; 3 Department of Surgery, Hiroshima City Hiroshima Citizens Hospital, Hiroshima, Japan; 4 Center for Innovative Clinical Medicine, Okayama University Hospital, Okayama, Japan; 5 Molecular Imaging Branch, Center for Cancer Research, National Cancer Institute, National Institutes of Health, Bethesda, MD USA

**Keywords:** Cancer microenvironment, Oesophageal cancer, Cancer immunotherapy

## Abstract

Cancer-associated fibroblasts (CAFs) play a significant role in tumor progression within the tumor microenvironment. Previously, we used near-infrared photoimmunotherapy (NIR-PIT), a next-generation cancer cell-targeted phototherapy, to establish CAF-targeted NIR-PIT. In this study, we investigated whether dual-targeted NIR-PIT, targeting cancer cells and CAFs, could be a therapeutic strategy. A total of 132 cases of esophageal cancer were analyzed for epidermal growth factor receptor (EGFR), human epidermal growth factor 2 (HER2), and fibroblast activation protein (FAP) expression using immunohistochemistry. Human esophageal cancer cells and CAFs were co-cultured and treated with single- or dual-targeted NIR-PIT in vitro. These cells were co-inoculated into BALB/c-*nu/nu* mice and the tumors were treated with single-targeted NIR-PIT or dual-targeted NIR-PIT in vivo. Survival analysis showed FAP- or EGFR-high patients had worse survival than patients with low expression of FAP or EGFR (log-rank, *P* < 0.001 and *P* = 0.074, respectively), while no difference was observed in HER2 status. In vitro*,* dual (EGFR/FAP)-targeted NIR-PIT induced specific therapeutic effects in cancer cells and CAFs along with suppressing tumor growth in vivo, whereas single-targeted NIR-PIT did not show any significance. Moreover, these experiments demonstrated that dual-targeted NIR-PIT could treat cancer cells and CAFs simultaneously with a single NIR light irradiation. We demonstrated the relationship between EGFR/FAP expression and prognosis of patients with esophageal cancer and the stronger therapeutic effect of dual-targeted NIR-PIT than single-targeted NIR-PIT in experimental models. Thus, dual-targeted NIR-PIT might be a promising therapeutic strategy for cancer treatment.

## Introduction

Esophageal cancer (EC), a highly malignant cancer, ranks sixth among all cancers in terms of mortality^1^. There are two main esophageal cancer subtypes: esophageal squamous cell carcinoma (ESCC) and esophageal adenocarcinoma (EAC), which comprise up to 90% of esophageal cancer cases worldwide. The incidence of EAC is increasing and has surpassed ESCC in several areas in Europe and North America^2^. Despite recent advances in diagnostics and therapeutics, the prognosis of EC remains poor, with a 5-year survival rate being approximately 15–20%^3,4^. Although several clinical trials have been conducted to develop novel therapies using molecular targeted drugs, such as the ErbB tyrosine kinase receptor family ErbB1/epidermal growth factor receptor (EGFR) or ErbB2/human epidermal growth factor 2 (HER2)^5,6^, the treatment results have been unsatisfactory^7,8^. Thus, there is a need to better understand the pathogenesis of EC to develop novel targeted therapies.

Tumor cell-extrinsic factors in the tumor microenvironment (TME) play a key role in tumor progression, invasion, and metastasis^9^. Cancer-associated fibroblasts (CAFs) are one of the major components of the TME and play a central role in tumor progression in various carcinomas, including colon, pancreatic, and breast cancers^10^. Previously, we reported that CAFs contribute to tumor progression by angiogenesis^11^, therapeutic resistance^12^, tumor immunosuppression^13^, and lymph node metastasis^14^ in patients with EC. Fibroblast activation protein (FAP) expression is highly specific and have a property of tumor promoting in various types of tumors, although CAFs are known to have heterogeneity and have various specific markers, including alpha smooth muscle actin (αSMA), vimentin, platelet-derived growth factor receptor (PDGFR), and fibroblast specific protein-1 (FSP-1)^15,16^. Thus, FAP-targeted therapy has been focused on and developed recently^17–21^.

Near-infrared photoimmunotherapy (NIR-PIT) is a recently developed as a next-generation cancer cell targeted phototherapy that causes cytotoxicity by specifically binding antibody-photosensitizer conjugate (APC) to target cells after irradiated NIR light^22^. As the mechanisms of NIR-PIT are gradually being revealed, this strategy can be applied to treat various cancer cells using various antibodies^22–30^. Presently, NIR-PIT against locoregional, recurrent head and neck squamous cell carcinoma (SCC) is under investigation in an international phase III clinical trial (LUZERA-301, NCT03769506). Moreover, the first APC (ASP-1929, Akalux™, Rakuten Medical Inc., San Diego, CA, USA) targeting EGFR and utilizing a NIR laser system (BioBlade™, Rakuten Medical Inc.) was conditionally approved for clinical use in September 2020 in Japan. TME-targeted NIR-PIT, especially novel therapies targeting CAFs, are attracting attention. Since we elucidated that FAP expression is associated with poor prognosis in patients with EC^14^, we applied NIR-PIT technology to establish NIR-PIT targeting FAP, a specific CAF marker, and successfully revealed its effect on tumor regression^12,31^. Finally, we hypothesized that targeting cancer cells and the TME using dual antibodies would be the most efficient treatment.

In this study, we further assessed whether dual-targeted NIR-PIT, targeting cancer cells and CAFs, could have a stronger therapeutic effect than single-targeted NIR-PIT, targeting cancer cells or CAFs, in EC patients. Therefore, we first performed clinicopathological analysis using primary tissue samples and then verified the effect of dual-targeted NIR-PIT using in vitro and in vivo models.

## Materials and methods

### Cell lines

In this study, we used human cell lines of esophageal squamous cell carcinoma (Tn, TE1, 4, 6, 8, 10, 13, 14, 15), esophageal adenocarcinoma (OE19, 33), fetal esophageal fibroblast 3 (FEF3), and GFP-FEF3. The details of these fibroblasts have been previously reported^11,14^. Human oral squamous cell carcinoma cells (HSC-2) were used in western blotting (WB) as an EGFR positive control and human breast cancer cells (SR-BK-3) as HER2 positive.

All cancer cell lines were purchased from the Japanese Collection of Research Bioresources (JCRB) Cell Bank (Osaka, Japan), and TE8 was purchased from the RIKEN BRC Cell Bank (Tsukuba, Japan). All cells were maintained at 37 °C in a 5% (vol/vol) CO_2_ incubator.

### Antibodies and reagents

The following antibodies were used in this study: monoclonal anti-epidermal growth factor receptor 1 (ErbB1/EGFR) antibody (Cell Signaling Technology, #4267), monoclonal anti-ErbB2 (ErbB2/HER2) antibody (Cell Signaling Technology, #4290) for WB and immunohistochemistry (IHC), and anti-β-actin (Sigma-Aldrich, A5441) for WB; monoclonal anti-αSMA antibody (Sigma-Aldrich, A5228) for IHC; and monoclonal anti-FAP antibody (R&D Systems, MAB3715) for the IR700-conjugated procedure. NHS ester of IR700 was purchased from LI-COR Biosciences (NE, USA). Trastuzumab and panitumumab were purchased from Chugai and Takeda Pharmaceutical Co., respectively.

### Activation of fibroblasts by conditioned media from esophageal cancer cells

Based on our previous study^31^, fibroblasts were activated by conditioned medium (CM), which is equivalent to activation in co-culture with cancer. To prepare the CM (CM/Cancer, e.g. CM/TE8), esophageal cancer cells were cultured in T175 flasks with Dulbecco’s modified Eagle’s medium (DMEM) containing 10% fetal bovine serum (FBS) and 1% penicillin–streptomycin (Sigma-Aldrich, MO, USA). After 24 h, the medium was replaced with DMEM containing 2% FBS and 1% penicillin–streptomycin, and the cells were incubated. After culturing for 48 h, the medium of CM/cancer cells was collected and centrifuged at 1000 rpm (same as 1071 G) for 5 min. This supernatant was collected as CM/Cancer, then This CM was stored at − 30 °C until use.

FEF3 cells were cultured in DMEM containing 10% FBS. After 24 h, the medium was replaced with CM/Cancer and incubated for 48–72 h, and the cells were defined as CAFs.

### Western blotting

The expression of EGFR and HER2 in esophageal cancer cells was examined using WB. These cells were lysed using cell lysis buffer (50 mmol/L Tris–HCl (pH 7.4), 30 mmol/L NaCl, and 1% Triton X-100) containing protease inhibitors (cOmplete Mini, Roche Diagnostics GmbH, Basel, Switzerland), and whole proteins extracted by centrifugation (14.0 × 10^3^ rpm same as 15.0 × 10^3^ G) for 10 min at 4 °C. The concentrations of extracted protein were measured using standard protocols. Samples containing 40 µg protein were electrophoresed on polyacrylamide gels and transferred to Hybond-polyvinylidene difluoride transfer membranes (GE Healthcare, UK). The membrane was incubated overnight at 4 °C with primary antibodies against EGFR (1:1000 dilution), HER2 (1:1000 dilution), and β-actin (1:1000 dilution). Membranes were washed in Tris Buffered Saline with Tween (TBST) and were incubated with secondary antibodies for 1 h at room temperature (RT) (20–22 °C). After washing, the membranes were visualized using an LAS-4000 mini (FUJIFILM, Tokyo, Japan).

### Synthesis of IR700-conjugated panitumumab, trastuzumab, and anti-FAP antibody

The conjugation of dyes with monoclonal antibodies (mAbs) has been reported previously^32^. In brief, panitumumab, trastuzumab, or anti-FAP antibody was incubated with IRDye700DX.

NHS ester (IR700) (LI-COR Bioscience, 66.8 μg, 34.2 nmol, 5 mmol/L in DMSO) in 0.3 mol /L Na_2_HPO_4_ (pH 8.5) at RT for 1 h (for panitumumab and trastuzumab 1000 μg) or for 2 h (for FAP 1000 μg). The mixture was purified on a Sephadex G50 column (PD-10; GE Healthcare, UK). Protein concentration was determined using a Bio-Rad protein assay kit (Bio-Rad, CA, USA) by measuring the absorption at 595 nm with spectroscopy. The IR700 concentration was measured by absorption at 689 nm using a UV–Vis spectrophotometer (Thermo Fisher Scientific, MA, USA). The number of fluorophore molecules per mAbs were adjusted to approximately 2, and APCs were defined as Pan-IR700, Tra-IR700, and FAP-IR700.

### In vitro cancer cell-, CAF- and dual-targeted NIR-PIT

Quantitative evaluation of cell viability was performed using the Cell Proliferation Kit II (XTT) (Roche Molecular Biochemicals, Mannheim, Germany), according to the manufacturer’s protocol. In cancer cell-targeted NIR-PIT, cancer cells were seeded in 96-well plate at 3.0 × 10^3^ cells per well and incubated for 24 h. The medium was replaced with fresh medium containing 20 μg/mL of Pan- or Tra-IR700 and incubated for 6 h at 37 °C. In dual-targeted NIR-PIT, cancer cells and FEF3 cells were seeded in 96-well plates at a density of 3 × 10^3^ cells/well total and co-cultured for 24 h. To stimulate FEF3, the medium was changed to CM/cancer and co-cultured for another 72 h. The cells were treated with the following APCs: (1) no treatment (control); (2) 20 μg/mL of Pan- or Tra-IR700; (3) 20 μg/mL of FAP-IR700; (4) 20 μg/mL of Pan- or Tra-IR700 and FAP-IR700, and incubated for 6 h at 37 °C. In all wells, the medium was replaced with fresh medium after the antibody reaction. Cells were irradiated with a red light-emitting diode (LED), which emits light at a wavelength of 670–710 nm (L700-05AU 700 nm; Epitex Co, Kyoto, Japan), and a power density of 15 or 25 mW/cm^2^, as measured with an optical power meter (PM 100, Thorlabs, Inc., NJ, USA). Cell viabilities were evaluated after incubation for 1 h at 37 °C after irradiation.

### Fluorescence microscope for cancer-, and dual-targeted NIR-PIT

In cancer cell-targeted NIR-PIT, cancer cells were seeded in 96-well plate at 3.0 × 10^3^ cells per well and incubated for 24 h. The medium was replaced with fresh medium containing 20 μg/mL of Pan- or Tra-IR700 and incubated for 6 h at 37 °C. In dual-targeted NIR-PIT, FEF3 cells were seeded in 96-well plates at 2.0 × 10^3^ cells/well and incubated for 24 h. After overnight attachment, the medium was replaced with CM/cancer cells, and cells were cultured for 72 h. Cancer cells labeled with Cell Tracker Blue CMAC dye (Thermo Fisher Scientific, MA, USA) were added at 5.0 × 10^3^ cells/well and co-cultured with CAFs for 12 h. These cells were treated 20 μg/mL of APC for 6 h at 37 °C as described above. After treatment, cells were washed with PBS and media with propidium iodide (PI, 1:2000 dilution) (Sigma-Aldrich, St Louis, MO, USA) were added to identify dead cells. Cells were then irradiated with NIR light (20 J/cm^2^), and morphological changes were observed before treatment and after incubation for 1 h at 37 °C after treatment using a fluorescence microscope (IX83; Olympus, Tokyo, Japan).

### In vivo dual-targeted NIR-PIT

Female athymic nude mice (BALB/c-*nu/nu*) were purchased from CLEA (Tokyo, Japan). Six-week-old female BALB/c-*nu/nu* mice were used in this study. TE8 or TE4 cells (3.0 × 10^6^ cells) and CAFs (6.0 × 10^6^ cells) were suspended in PBS (50 μL) and basement membrane matrix (100 μL) (BD Biosciences, NJ, USA) and injected subcutaneously into the right flank. When the tumor reached 100 mm^3^ after injection, the mice were randomized into four groups: (a) no treatment group (control); (b) treatment groups by intraperitoneal (i. p.) injection^33^ with 50 μg/body of pan- or Tra-IR700 (cancer-PIT); (c) 50 μg/body of FAP-IR700 (CAF-PIT); (d) 50 μg/body of pan- or Tra-IR700 plus FAP-IR700 (dual-PIT). In order to focus on the therapeutic effect of dual-targeted NIR-PIT, the dose of antibody used in each group was 50 μg, half of our previous dose^12^. In the treatment groups, APC was injected on days 0 and 7, and then the mice were irradiated with NIR light at 50 J/cm^2^ on day 1–2, 8–9, respectively. Tumor diameter and body weight were measured three times per week. Tumor volume (mm^3^) was calculated using the formula as follows: length × width^2^ × 0.5.

### Patients and clinical information

A total of 149 patients who underwent esophagectomy at the Department of Gastroenterological Surgery of Okayama University Hospital between 2008 and 2010 were enrolled in this retrospective study. The following patients were excluded: (1) those who had undergone endoscopic mucosal resection (EMR) or endoscopic submucosal dissection (ESD) followed by surgery, (2) those who were diagnosed with melanoma or distant metastasis, (3) those who had no tumor presence/complete response after neoadjuvant therapy, and (4) those who were difficult to evaluate. Totally, 132 patients were included in the analysis. All patients were reviewed for age, sex, tumor depth (pT), lymph node status (pN), histological type, and neoadjuvant therapy. Tumor classification and staging were performed according to the TNM Classification of Malignant Tumors 7th edition (UICC 7th edition)^34^. The use of clinical samples was approved and reviewed by the ethics review board of Okayama University, Okayama, Japan (No. 1801–023).

### Immunohistochemical analysis

Formalin-fixed and paraffin-embedded surgical specimens of esophageal carcinoma were examined. Tissue sections were then deparaffinized and soaked in 0.3% H_2_O_2_ in methanol to eliminate endogenous peroxidase activity at RT for 10 min.

For FAP, antigen retrieval was performed by heating the specimens in sodium citrate buffer solution using microwaves. After cooling, the sections were incubated with a primary antibody against FAP for 60 min at RT. The details of staining for FAP have been previously reported^14^. For EGFR or HER2, antigen retrieval was performed by heating the specimens in ethylenediaminetetraacetic acid using microwaves. After cooling, the sections were incubated with a primary antibody against EGFR or HER2 overnight at 4 °C. After incubation with a primary antibody, sections were incubated with anti-rabbit (for FAP/EGFR/HER2) antibody as a secondary antibody for 30 min at RT. The enzyme substrate was 3,3′-diaminobenzidine (Dako) for visualization, and the sections were counterstained with Meyer’s hematoxylin.

### Evaluation of FAP/EGFR/HER2 score

First, the presence of tumor tissue was confirmed by hematoxylin and eosin (HE) staining. FAP scores were evaluated by combining the proportion and intensity scores, as previously described^14^. Briefly, the proportion score was assessed semi-quantitatively using ImageJ software (http://rsb.info.nih.gov/ij/https://imagej.nih.gov/ij/https://imagej.nih.gov/ij/), and the intensity score was graded visually. The scoring of EGFR and HER2 was performed according to the Trastuzumab for Gastric cancer (ToGA) study^35^. In this study, EGFR and HER2 expression level of 2 + or 3 + was defined as high, whereas that of 0 or 1 + was low. Histological assessments were performed by two independent pathologists who were blinded to the clinical data.

### Statistical analysis

All statistical analyses were performed using the JMP software (SAS Institute, NC, USA). For two-group comparisons *of *in vitro and in vivo experiments, the Mann–Whitney test or Student’s *t* test was used. For multiple group comparisons, analysis of variance with Tukey’s test was used. The correlation of FAP, EGFR, or HER2 expression with clinicopathological variables was evaluated using Pearson’s chi-squared test, Fisher’s exact test, or Student’s *t* test, as appropriate. Overall survival (OS) and disease-free survival (DFS) were calculated using the Kaplan–Meier method, and the log-rank test with Bonferroni correction was used to compare subgroups. Hazard ratios (HRs) and 95% confidence intervals (CIs) for clinical variables were calculated using Cox proportional hazards regression in the univariate and multivariate analyses. Statistical significance was set at* P* < 0.05.

### Study approval

This study was conducted in accordance with the ethical standards of the Helsinki Declaration and the ethical guidelines for medical and health research involving human subjects^36^. All cases were de-identified and details were removed from the case descriptions to ensure anonymity. The outline of our study was published on our webpage to explain the study and provide opportunities for disagreement. Owing to its retrospective nature, we requested and received permission to waive informed consent from the ethics committee. The use of clinical samples was approved and reviewed by the ethics review board of Okayama University (No. 1801–023; Okayama, Japan). Mouse experiments were conducted in a specific pathogen-free environment at the Okayama University Animal Facility in accordance with the institutional guidelines, and all animal experimental protocols were approved and reviewed by the Ethics Review Committee for Animal Experiments at Okayama University. All experiments were conducted in accordance with the guidelines and regulations of the committee.

### Ethics approval

The use of clinical samples was approved and reviewed by the ethics review board of Okayama University (No. 1801-023; Okayama, Japan). All animal experimental protocols were approved and reviewed by the Ethics Review Committee for Animal Experiments at Okayama University. All experiments were conducted in accordance with the guidelines and regulations of the committee. The study is reported in accordance with ARRIVE guidelines.

### Consent to participate

Owing to its retrospective nature, we requested and received permission to waive informed consent from the ethics review board of Okayama University.

## Results

### Esophageal cancer patients with high EGFR/FAP expression demonstrated poor prognosis

**Figure 1:**
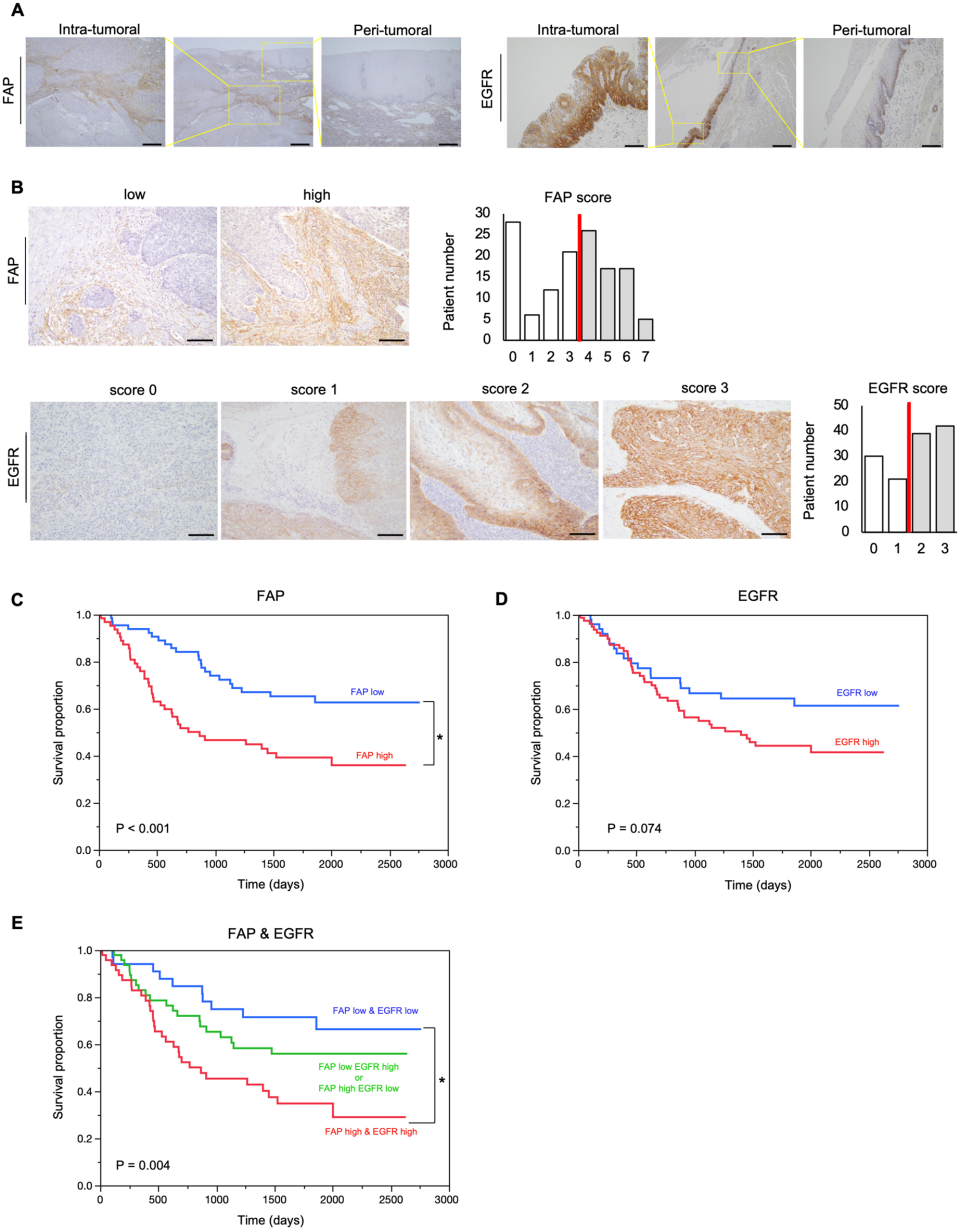
The expression of FAP and EGFR and clinical outcome in 132 cases of esophageal cancer. (**A**) IHC for FAP and EGFR are shown at low and high magnification (40 × and 200 ×). Scale bars: 500 µm (40 ×), 100 µm (200 ×). The expression of FAP and EGFR is different between intra-tumoral and peri-tumoral tissue. (**B**) Representative example of a low- and high-FAP and EGFR case. Scale bars: 200 µm (100 ×). The FAP score of 4 + or more were defined as high, that of 3 + or less were low; the EGFR expression level of 2 + or 3 + were defined as high, that of 0 or 1 + were low. (**C**) Survival analysis showed that FAP high patients had significantly worse OS than those with low FAP (*P* < 0.001, log-rank test; *P < 0.05). (**D**) Patients with high EGFR tended to show poorer OS than those with low EGFR (*P* = 0.074, log-rank test). (**E**) Survival curve of three groups divided by combination of EGFR and FAP scores (double negative, single positive, double positive), double positive group had worse survival than others (*P* = 0.005: vs. double negative, *P* = 0.089: vs. single positive, log-rank test; *P < 0.05).

To explore the expression of EGFR, HER2, and FAP in esophageal cancer, we conducted IHC using surgically resected tissues. Representative images are shown in Fig. 1A and Supplementary Fig. S1A. Patients were divided into high and low groups according to each protein expression described above. The population of each group was as follows: FAP low/high cases were 67 (50.8%)/65 (49.2%), EGFR low/high cases were 51 (38.6%)/81 (61.4%), and HER 2 low/high cases were 115 (87.1%)/17 (12.9%) (Fig. 1B*,* Supplementary Fig. S1B, Supplementary Table S1). Survival analysis showed that FAP-high patients had significantly worse OS than those with low FAP levels (log-rank, *P* < 0.001) (Fig. 1C, Supplementary Fig. S1C). Although patients with high EGFR expression tended to have poorer OS than those with low EGFR expression, EGFR expression was not a significant prognostic factor (log-rank, *P* = 0.074) (Fig. 1D). When analyzing the survival of the three groups divided by the combination of EGFR and FAP scores (double negative, single positive, double positive), the double-positive group had significantly worse survival than the other groups (log-rank, *P* = 0.002: vs. double negative, *P* = 0.030: vs. single positive) (Fig. 1E). However, no difference was observed between patients with high HER2 and those with low HER2 expression in the analysis of OS (log-rank, *P* = 0.361) (Supplementary Fig. S1D). Among the three groups divided by the combination of HER2 and FAP scores, the single positive group and double positive groups had significantly worse survival than the double negative group (log-rank, *P* = 0.006: single positive vs. double negative, *P* < 0.001: double positive vs. double negative) (Supplementary Fig. S1E).

In the univariate analysis for OS, sex, tumor depth, lymph node status, and high FAP expression were statistically significant prognostic factors. Furthermore, sex, tumor depth, lymph node status, high FAP expression, and high EGFR expression were significant prognostic factors for DFS (Supplementary Table S2). Thus, high expression of both FAP and EGFR strongly contributed to the prognosis of esophageal cancer. Although additional analysis was limited to squamous cell carcinoma, taking into account the prognostic differences between squamous cell carcinoma and adenocarcinoma, the results were comparable. (Supplementary Fig. S5, Supplementary Tables S3, S4).

### NIR-PIT against EC cells had a dramatic anti-tumor effect

**Figure 2:**
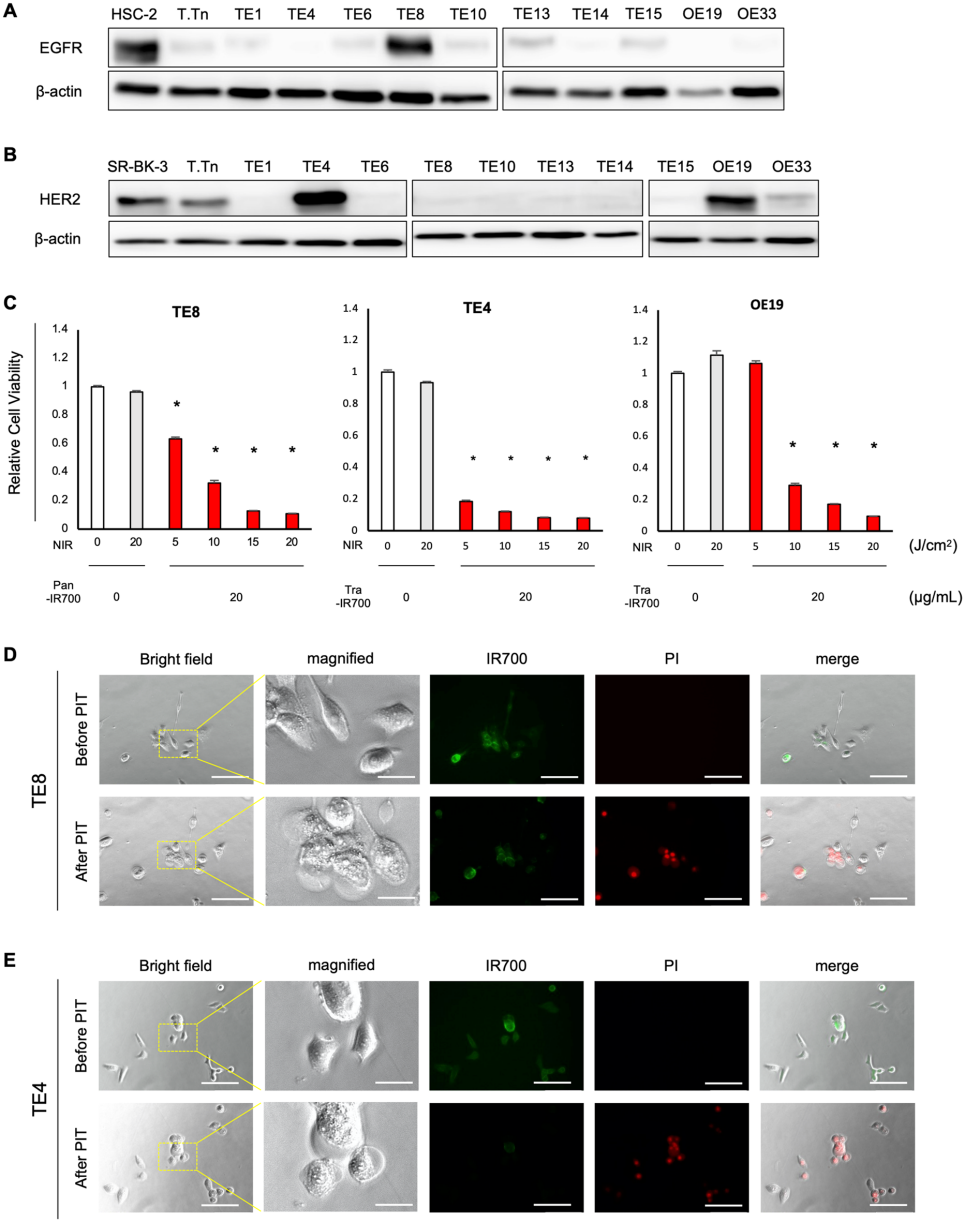
Cancer cell-targeted NIR-PIT for esophageal cancer cells in vitro. (**A,B**) Western blots showed that TE8 cells was EGFR strongly positive, TE4 and OE19 cells were HER2 strongly positive. (**C**) TE8 cells were treated with Pan-IR700 (20 μg/mL), TE4 and OE19 cells were treated with Tra-IR700 (20 μg/mL). The cytotoxic effect was dependent on the dose of NIR light, although NIR light irradiation alone showed no cytotoxic effect. n = 3; mean ± SEM; ^*^P < 0.05, Student’s *t* test (compared to untreated control). (**D,E**) Microscopic images. Co-cultured cells were stained for mAb-IR700 (IR700; green) and dead cells (PI; red). Immunofluorescent microscopy showed that Pan-IR700 (**D**) or Tra-IR700 (**E**) bound to EGFR or HER2 before NIR-PIT. After NIR irradiation, bleb formation and cell swelling were observed on the target cell. PI staining was also confirmed consistent with this change. Scale bars: 50 μm, 25 μm (magnified).

We confirmed the expression of EGFR and HER2 in EC cell lines by WB. WB demonstrated that TE8 cells strongly expressed EGFR, whereas TE4 and OE19 cells had high HER2 expression (Fig. 2A–B). NIR-PIT against each target molecule was performed in vitro. TE8 cells were treated with Pan-IR700 (20 μg/mL), TE4 and OE19 cells were treated with Tra-IR700 (20 μg/mL), and then these cells were irradiated with NIR light. Most cells were killed by NIR-PIT targeting EGFR or HER2. The cytotoxic effect was dependent on the dose of the NIR light (Fig. 2C, Supplementary Fig. S2A); however, there is no change in cytotoxicity after the amount of antibody exceeds 20 μg/mL (data not shown). NIR light irradiation alone showed no cytotoxic effects (data not shown). Morphological changes were observed before and after NIR-PIT (Fig. 2D–E). Immunofluorescence microscopy showed that Pan-IR700 or Tra-IR700 bound to EGFR or HER2 before NIR-PIT. After NIR irradiation, immediate bleb formation was observed on the cell membrane and the cancer cells were completely swollen and disintegrated 1 h after NIR irradiation; PI staining was also confirmed to be consistent with this change. No therapeutic effect was observed in cells weakly positive or negative for EGFR or HER2 expression (Supplementary Fig. S2B).

### Dual-targeted NIR-PIT against EC cells and CAFs induced specific therapeutic effects in each cell type

**Figure 3:**
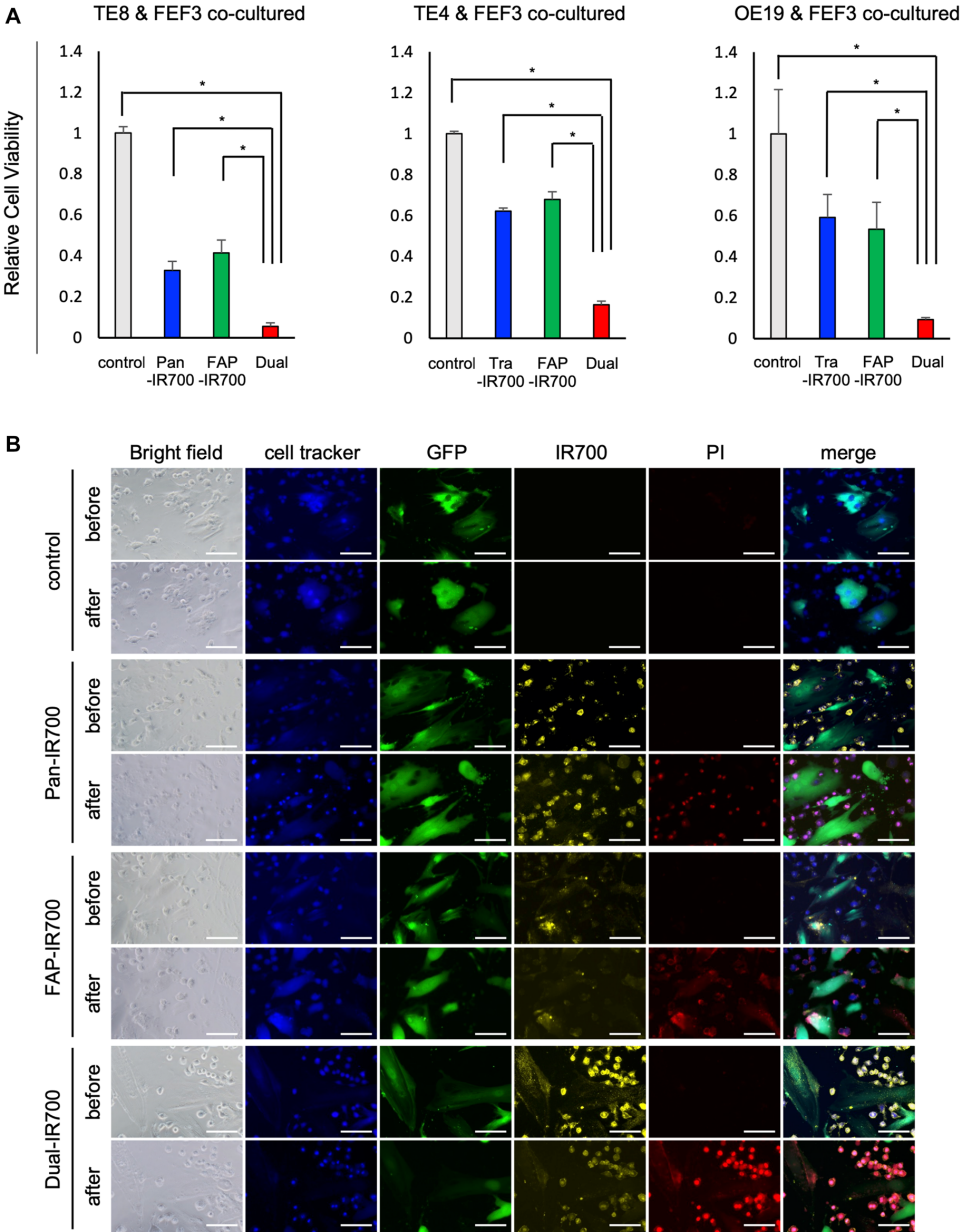
Dual-targeted NIR-PIT for esophageal cancer cells and CAFs in vitro. (**A**) Selectivity of APC targeted to each cell and the influence on adjacent cells under co-culture of cancer cells and fibroblasts were investigated (n = 3, in each group). Dual-targeted NIR-PIT induced stronger cytotoxicity compared to each single NIR-PIT under a co-culture of cancer cells (TE8, TE4 or OE19) and FEF3. Dual-Targeted NIR-PIT showed an additive effect, confirming that cancer cells and CAFs could be treated simultaneously with a single NIR light irradiation. (mean ± SEM. *P < 0.01; Student’s *t* test). (**B**) Immunofluorescent microscopic images presenting NIR-PIT under co-culture of TE8 and FEF3 cells. In the bright field, TE8 cells are recognized as round cells, morphologically distinct from spindle-shaped FEF3 cells. Cultures were stained for cancer cells (cell tracker; blue), GFP-FEF3 cells (GFP; green), mAb-IR700 (IR700; yellow), and dead cells (PI; red). PI was not observed in untargeted cells or adjacent cells in Single- (Cancer cell- or CAF-) targeted NIR-PIT under co-cultivation. Dual-targeted NIR-PIT demonstrated that cancer cells and CAFs were treated simultaneously with a single NIR light irradiation. Scale bars: 50 μm.

We have previously reported the effect of NIR-PIT on CAFs^31^. Furthermore, no therapeutic effect was observed in cells negative for FAP expression (data not shown). Next, we investigated the selectivity of APCs targeted to each cell and their influence on surrounding cells under co-culture of cancer cells and fibroblasts. Pan-IR700 (20 μg/mL) was used for TE8 cells, Tra-IR700 (20 μg/mL) for TE4 and OE19 cells, and FAP-IR700 (20 μg/mL) for CAFs. After the reaction, NIR light (20 J/cm^2^) was irradiated. Each APCs had high selectivity to the target cells and no cross reactivity was observed (data not shown). It was confirmed dual-targeted NIR-PIT induced stronger cytotoxicity compared to each single NIR-PIT under a co-culture of cancer cells (TE8, TE4, or OE19) and FEF3 cells (Fig. 3A). Immunofluorescence microscopy showed that PI was not observed in untargeted cells or adjacent cells in single- (cancer cell- or CAFs-) targeted NIR-PIT under co-cultivation (Fig. 3B, Supplementary Fig. S3A–B). Dual-targeted NIR-PIT showed an additive effect, confirming that cancer cells and CAFs can be treated simultaneously with a single NIR irradiation.

### Dual-targeted NIR-PIT inhibited tumor growth in vivo

**Figure 4:**
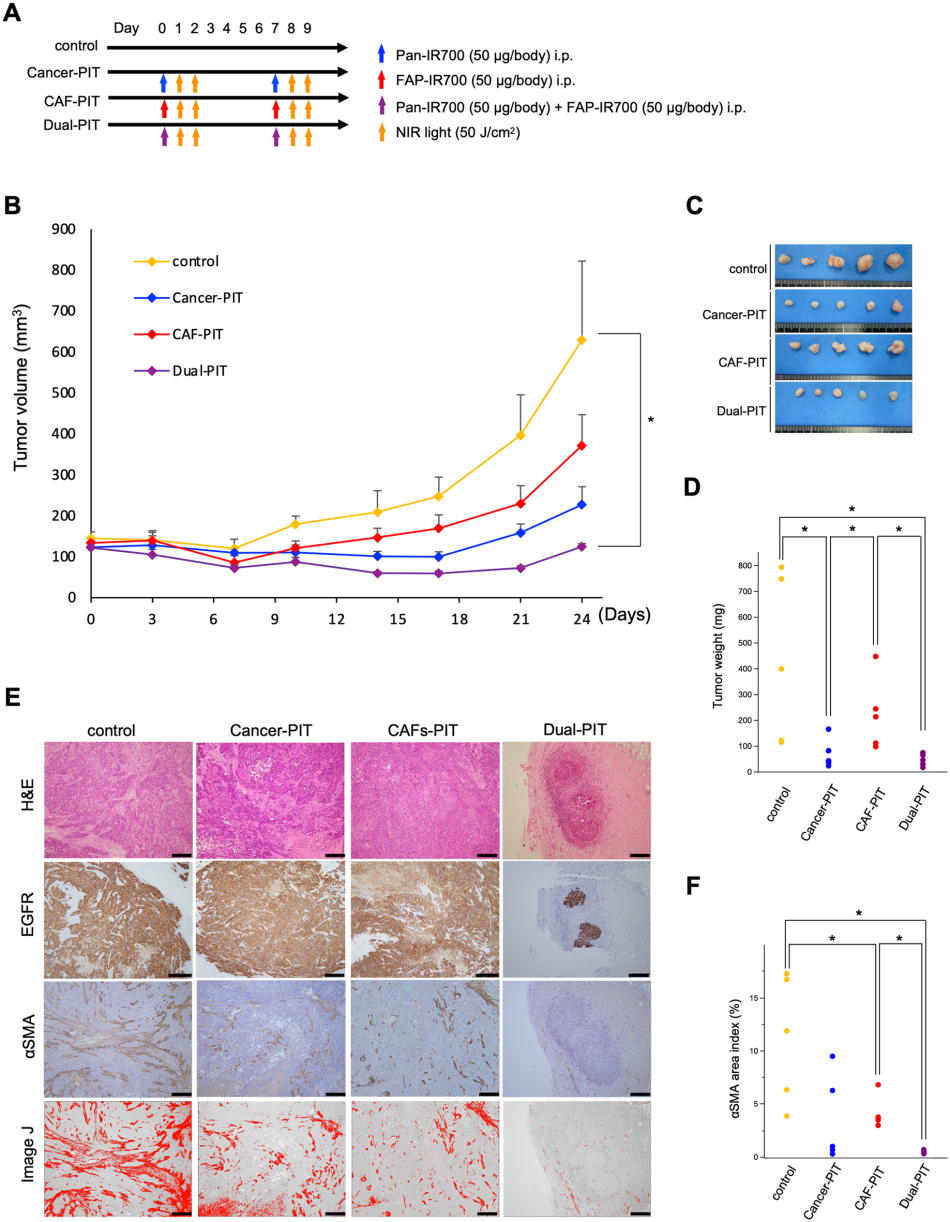
Dual-targeted NIR-PIT for TE8 cells and CAFs in vivo. (**A**) Treatment protocol. (**B**) Tumor growth of subcutaneous tumors inoculated in BALB/c-*nu/nu* mice (n = 5, in each group). The tumors were dramatically suppressed in the Dual-PIT group compared with the control group (mean ± SEM. *P < 0.05, Tukey’s test with ANOVA). (**C,D**) Evaluation of tumor weight is shown for each group (*P < 0.05, Tukey’s test with ANOVA). (**E**) IHC of EGFR and αSMA in each group. The amount of fibroblasts was quantified by Image J. Scale bars: 400 µm (100 ×). (**F**) Quantification of the αSMA area index is shown for each group (*P < 0.05, Tukey’s test with ANOVA).

**Figure 5:**
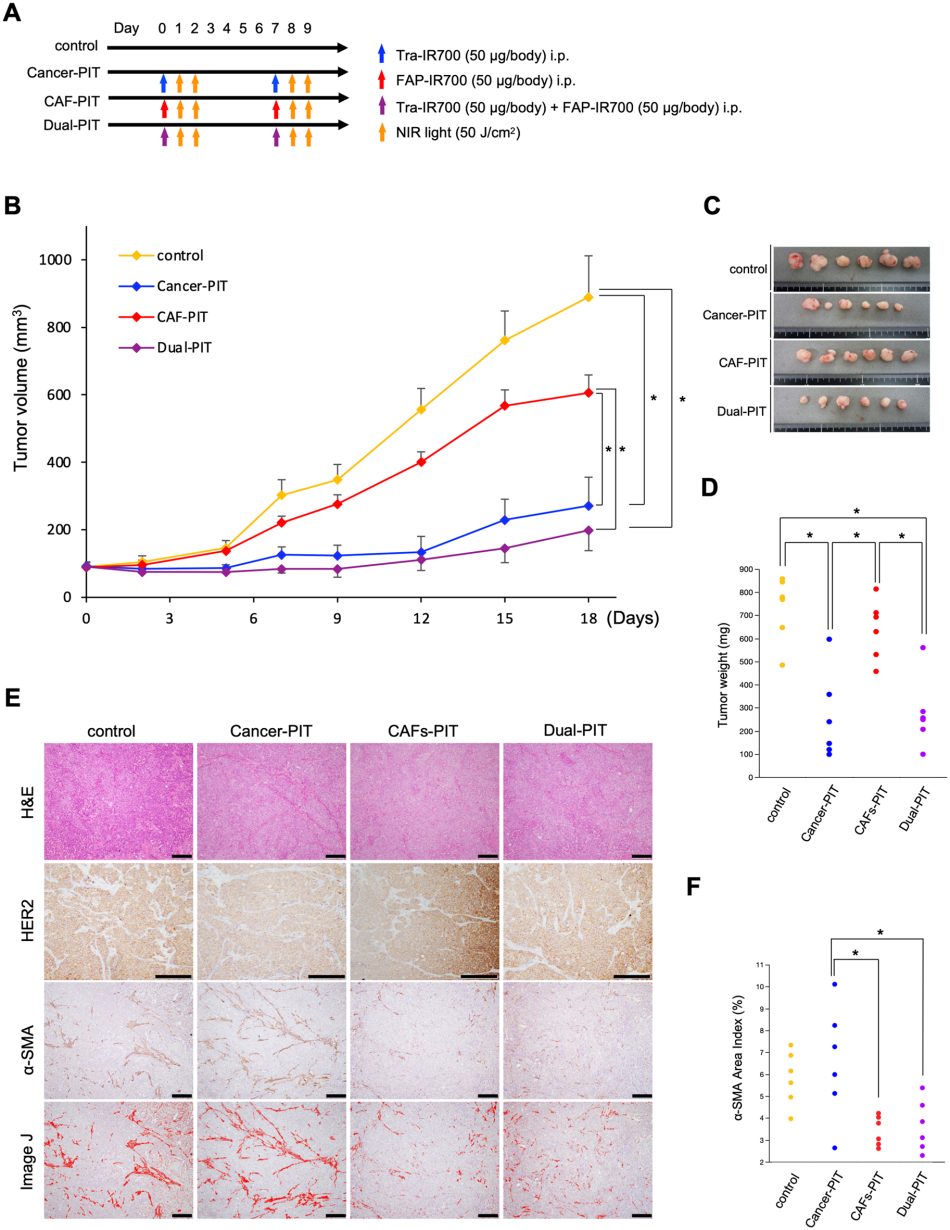
Dual-targeted NIR-PIT for TE4 cells and CAFs in vivo. (**A**) Treatment protocol. (**B**) Tumor growth of subcutaneous tumors inoculated in BALB/c-*nu/nu* mice (n = 6, in each group). The tumors were dramatically suppressed in the dual-PIT group compared with the control group (mean ± SEM. *P = 0.018, Tukey’s test with ANOVA). (**C,D**) Evaluation of tumor weight is shown for each group (*P < 0.05, Tukey’s test with ANOVA). (**E**) IHC of EGFR and αSMA in each group. The amount of fibroblasts was quantified by Image J. Scale bars: 400 µm (100 ×). (**F**) Quantification of the αSMA area index is shown for each group (*P < 0.05, Tukey’s test with ANOVA).

First, we confirmed the effect of cancer cell-targeted NIR-PIT using Tra-IR700 in a xenograft model inoculated with TE4 cells in BALB/c-*nu/nu* mice (Supplementary Fig. S4). Next, to verify the effect of dual-targeted NIR-PIT, we compared the therapeutic effect of the following treatment groups using a xenograft model co-inoculated with TE8 cells and CAFs into BALB/c-*nu/nu* mice: (a) control, (b) cancer-PIT, (c) CAF-PIT, and (d) dual-PIT. In the treatment groups, APC was injected on days 0 and 7, and then the mice were irradiated with NIR light at 50 J/cm^2^ on day 1–2, 8–9, respectively (Fig. 4A). The results showed that the tumors were dramatically suppressed in the dual-PIT group compared to the control group (Fig. 4B,C). Although tumor suppression was observed in both the cancer-PIT and CAF-PIT groups compared with the control group, the difference was not statistically significant. Tumor weights were significantly decreased in the cancer-PIT and dual-PIT groups (Fig. 4D). None of the mice showed any side effects, such as skin disorders or body weight loss (data not shown). It should be noted that the number of fibroblasts was significantly reduced in the CAF-PIT and dual-PIT groups (Fig. 4E,F); thus, the network of fibroblasts was disrupted by CAF-PIT, which contributed to tumor regression. Similar trends were observed in the xenograft model co-inoculated with TE4 cells and CAFs; dual-targeted NIR-PIT using Tra-IR700 and FAP-IR700 also significantly suppressed the tumor growth (Fig. 5).

## Discussion

In this study, we demonstrated that cancer cell-targeted NIR-PIT utilizing pan-IR700 or tra-IR700 is effective against EGFR and HER2 positive EC cells and showed in vitro and in vivo the additive effect of dual-targeted PIT against EC cells and CAFs. Although the therapeutic effect of cancer cell-targeted PIT was remarkable in EC cells, tumor volume could be further reduced by dual-targeted NIR-PIT. Furthermore, to the best of our knowledge, this is the first study to report the correlation between EGFR-expressing cancer cells and FAP-expressing CAFs in EC; patients with high EGFR or FAP expression had poor prognosis, and patients with high expression of both EGFR and FAP had the worst survival. Our results suggest that dual-targeted therapy against cancer cells and CAFs may be more effective than single-targeted therapy for patients with high expression of both EGFR and FAP.

Although recent developments in targeted cancer therapeutics have enabled personalized medicine, clinical trials have failed to show the effectiveness of these therapies^37^. Practically, clinical trials evaluating the effect of adding EGFR- or HER2-targeted agents for EC did not show any benefit for survival^7,8^. Currently, there is a gap in the lack of effective treatments for a large amount of preclinical data, partly due to the high cost of clinical trials, limited patient access to available drugs, clinical trials, and the biological complexity of cancer. Further advances in cancer therapy will be associated with bioinformatic analyses to understand not only the dynamic changes in tumor biology, but also the surrounding TME. As the TME is known to be involved in acquired resistance of tumors to various therapies, CAFs, which play a key role in drug resistance, are considered candidate therapeutic targets^38^.

No proposed CAF-targeted therapy has proven to be effective in clinical applications^39^. Previously, therapies targeting CAFs have been developed in the following ways: (1) inhibiting the function of CAFs, (2) inhibiting their differentiation into CAFs, (3) inhibiting their communication with cancer cells, and (4) eliminating CAFs themselves. Although eliminating CAFs is the most reasonable approach and has been evaluated in clinical trials, no study has shown clinical effectiveness, which may be due in part to the heterogeneous nature of CAFs. Kobayashi et al. offered a novel nomenclature for CAFs based on the function of tumor-promoting CAFs, tumor-retarding CAFs, and neural CAFs; however, none of the various CAF markers could distinguish all CAFs^40^. Among them, FAP has been reported to be associated with expression and prognosis in EC, colorectal cancer, and pancreatic ductal adenocarcinoma^14,41,42^ and is a marker of tumor-promoting CAF. However, no FAP-targeted therapy has demonstrated efficacy in colorectal cancer patients, as both sibrotuzumab (a FAP-targeted mAb) and talabostat (a small molecule FAP-inhibitor) were incapable of successfully passing phase II trials^43,44^. In those studies, serious toxicity made it difficult to continue treatment, and this was considered to be an adverse reaction to targeting FAP, which is also expressed in normal cells throughout the body.

NIR-PIT is a recently developed next-generation molecular targeted therapy that employs a target-specific photosensitizer conjugated to mAbs targeting specific membrane molecules, inducing specific cell death after irradiated NIR light^22,45^. The strength of NIR-PIT lies in its ability to target any membrane molecule, not just a specific molecule, which may enable the application of molecularly targeted drugs that have not been previously effective. Furthermore, the target of NIR-PIT is not limited to cancer cells, allowing for a therapeutic strategy to target TMEs^46–48^. We have established NIR-PIT targeting CAFs using FAP mAbs and have demonstrated its tumor-suppressive effect without toxicity^31^. FAP-targeted therapy with NIR-PIT can specifically target FAP in the tumor microenvironment and is expected to have a high antitumor effect with less systemic toxicity.

Dual-targeted NIR-PIT is a highly reasonable strategy that can simultaneously attack both cancer cells and CAFs. CAFs promote tumor growth, contribute to a poor response to various therapies, such as chemotherapy, radiation, and immunotherapy, and induce tumor immunosuppression, leading to poor survival^49–51^. The additional tumor-suppressive effect of dual-targeted NIR-PIT over cancer cell-targeted NIR-PIT in vivo could be interpreted as the elimination of CAFs, which not only suppressed tumor growth signals but also improved drug delivery to the tumor, amplifying the effect of cancer cell-targeted NIR-PIT^26,52^. Although we did not evaluate the accompanying immunoreactions as our in vivo tumor models were established using immune-deficient mice, the immunostimulatory effect of CAF-targeted NIR-PIT is remarkably interesting. As we previously reported that CAFs modulated the intra-tumoral migration of TILs, the elimination of CAFs might amplify the effect of cancer cell-targeted NIR-PIT on immunogenic cell death (ICD)^13^. Furthermore, dual-targeted NIR-PIT offers the simplicity of simultaneous drug administration and irradiation for both cancer cells and CAFs, which is a highly reasonable treatment strategy.

Here, we examined the clinicopathological relationship between the ErbB tyrosine kinase receptor family and CAFs in clinical samples and demonstrated that both high FAP and EGFR expression strongly contributed to esophageal cancer prognosis. Previous studies have reported that EGFR signaling mediates cancer-stroma crosstalk and is associated with metastasis and recurrence^53,54^. In the present study, we found that EGFR is not only involved in the survival of esophageal cancer patients but is also associated with poor survival in association with FAP, which is a novel finding evaluating the association between EGFR signaling and the stroma in clinical specimens. Thus, dual-targeted NIR-PIT is a reasonable treatment for esophageal cancer patients with poor prognosis expressing both EGFR and FAP, as it can simultaneously attack both cancer cells and stroma and break the relationship.

Our study had several limitations. First, since the criteria for evaluating the expression of the ErbB tyrosine kinase receptor family in EC are not standardized^5^, we analyzed the data according to the guidelines for gastric cancer^35^. On the other hand, we believe that this study is valuable in that we evaluated the association between EGFR/HER2 expression and clinicopathological characteristics in more than 130 EC patients using a set of criteria. However, further studies are needed to establish methods for evaluating these biomarkers in EC, as the guidelines for assessing biomarkers vary between cancer types, such as breast cancer and gastric cancer^55^. Second, although we showed the potential of dual-targeted NIR-PIT in xenograft models using co-inoculation, the immunogenic reaction triggered by this potential therapy was not evaluated. We expect dual-targeted NIR-PIT to have immunogenic synergistic effects, such as ICD caused by cancer cell-targeted NIR-PIT and immune activation triggered by CAF-targeted NIR-PIT. To evaluate these immunologic consequences, the use of a syngeneic model is necessary. Third, we evaluated the treatment effect over a relatively short period of time. However, previous reports showed that the NIR-PIT treatment effects, evaluated within approximately 20 days, correlate with long-term results^22,56,57^. Accordingly, the same time frame was used in this study to assess therapeutic outcomes. However, it would be valuable to evaluate the long-term results, which would probably be similar. Fourth, this study did not characterized the distribution of APCs in vivo. Although previous studies have demonstrated specific APC binding to target cells, but there is no experience validating in stroma-rich tumors such as those in this study^22^. Therefore, the status of APC accumulation can be an issue for future study. Fifth, the total used amount of APCs was twice the concentration in the dual-PIT group. However, because of the high selectivity of each treatment and the verified lack of cross-reactivity, each APC did not affect each cell. Furthermore, we confirmed that doubling the amount of APC did not increase the cytotoxic effect in vitro experiments (data not shown). Therefore, this experiment demonstrated the usefulness of a treatment that simultaneously attacks cancer cells and CAFs with NIR-PIT.

In conclusion, we demonstrated the relationship between EGFR/FAP expression and the prognosis of EC patients. Additionally, the stronger therapeutic effect of dual-targeted NIR-PIT compared to that of single-targeted NIR-PIT was shown in both in vitro and in vivo models. Thus, dual-targeted NIR-PIT may be a promising therapeutic strategy for cancer treatment.

## Supplementary Information

Supplementary Information.

## Data Availability

The data that support the findings of this study are available from the corresponding author, KN, upon reasonable request.

## Abbreviations

AB: Antibody
AF: Activated fibroblast
APC: Antibody-photo-absorber conjugate
CAF: Cancer-associated fibroblast
CM: Conditioned medium
DAPI: 4,6-Diamidino-2-phenylindole
DMEM: Dulbecco’s modified eagle medium
EAC: Esophageal adenocarcinoma
ESCC: Esophageal squamous cell carcinoma
EGFR: Epidermal growth factor receptor
FACS: Fluorescence-activated cell sorting
FAP: Fibroblast activation protein
FBS: Fetal bovine serum
FEF3: Fetal esophageal fibroblasts
FSP1: Fibroblast-specific protein 1
GFP: Green fluorescent protein
HER2: Human epidermal growth factor 2
IHC: Immunohistochemistry
LED: Light-emitting diode
mAb: Monoclonal antibody
NIR: Near infrared
PBS: Phosphate-buffered saline
PDA: Pancreatic ductal adenocarcinoma
PIT: Photoimmunotherapy
RT: Room temperature
SCC: Squamous cell carcinoma
SMA: Smooth muscle actin
TBST: Tris buffered saline with tween
TGF: Transforming growth factor
TME: Tumor microenvironment
VEGF: Vascular endothelial growth factor
WB: Western blotting
XTT: Sodium 2,3,-bis(2-methoxy-4-nitro-5-sulfophenyl)-5-[(phenylamino)-carbonyl]-2H-tetrazolium
