# Supplementary material for: Dual-targeted near-infrared photoimmunotherapy for esophageal cancer and cancer-associated fibroblasts in the tumor microenvironment
Source: Sci Rep. 2022 Nov 23;12:20152. doi: 10.1038/s41598-022-24313-3 (PMC9684531; doi:10.1038/s41598-022-24313-3)

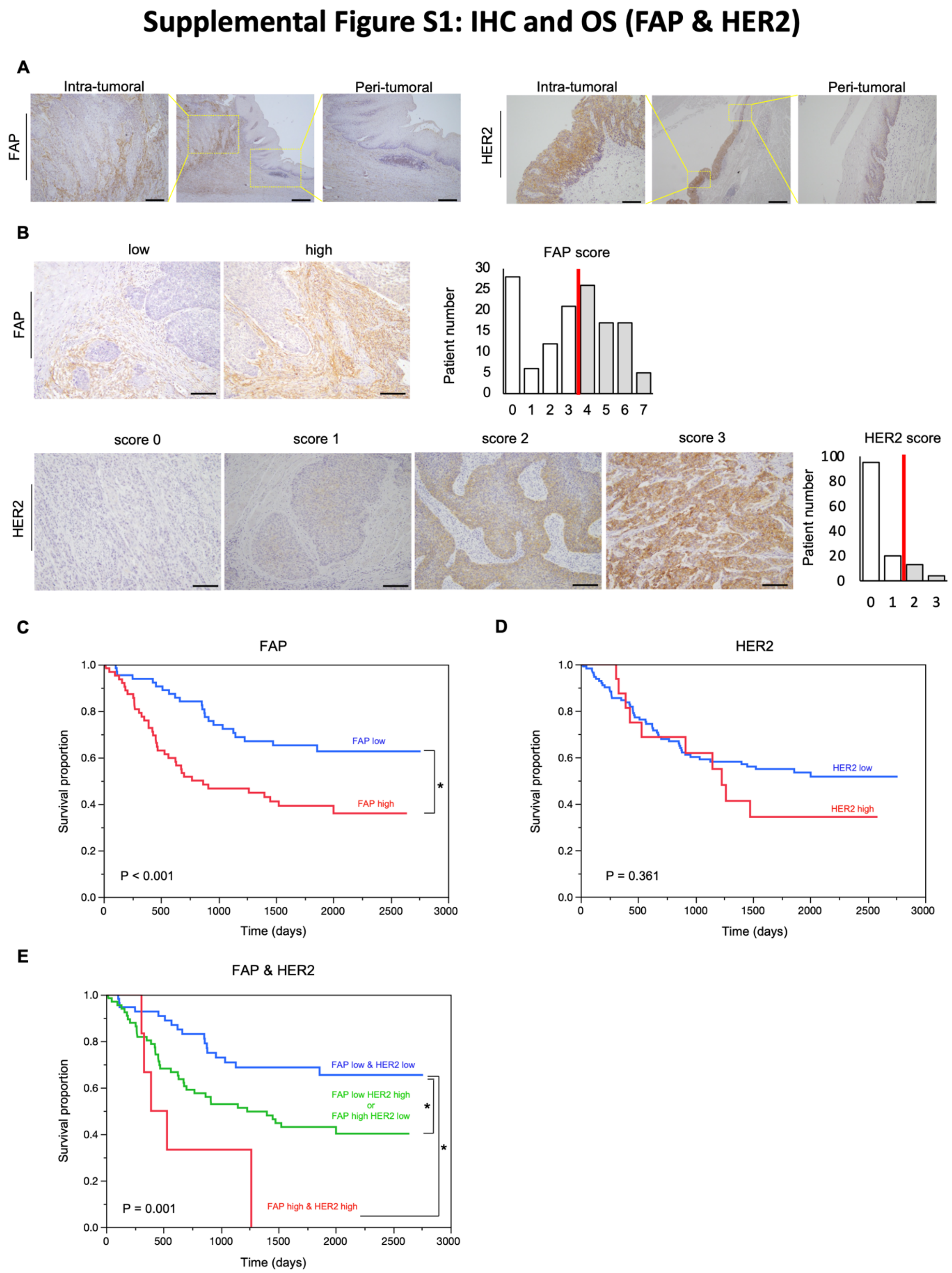

Supplemental Figure S1. The expression of FAP and HER2 and clinical outcome in 132 cases of esophageal cancer

(A) IHC for FAP and HER2 are shown at low and high magnification (FAP: 40× and 100×, HER2: 40× and 200×). Scale bars: 500 μm (40×), 200 μm (100×), 100 μm (200×). The expression of FAP and HER2 is different between intra-tumoral and peri-tumoral tissue. (B) Representative example of a low- and high- FAP and HER2 case. Scale bars: 200 μm (100×) The FAP score of 4+or more were defined as high, that of 3+ or less were low; the HER2 expression level of 2+ or 3+ were defined as high, that of 0 or 1+ were low.

(C) Survival analysis showed that FAP high patients had significantly worse OS than those with low FAP ($P < 0.001$, Log-Rank)

(D) No difference was observed between patients with high HER2 and those with low HER2 in the analysis for OS ($P = 0.361$, Log-Rank).

(E) Survival curve of three groups divided by combination of HER2 and FAP scores (double negative, single positive, double positive), single positive group and double positive group had worse survival than double negative group ($P = 0.006$: single positive vs double negative, $P < 0.001$: double positive vs double negative, log-rank test; *, $P < 0.05$)

Supplemental Figure S2: Cancer- targeted NIR-PIT in vitro

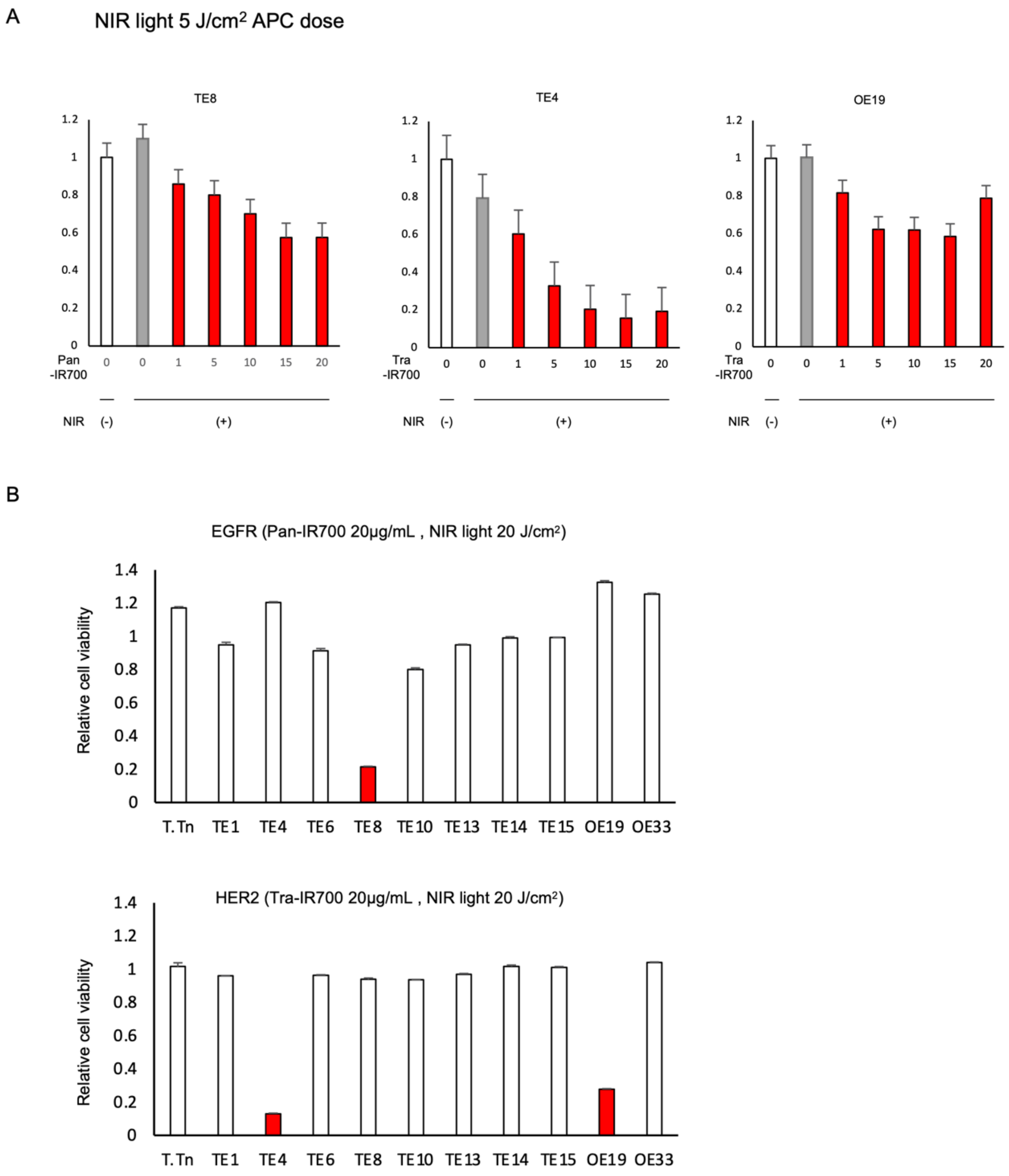

Supplemental Figure S2. Cancer- targeted NIR-PIT in vitro

(A) The cytotoxic effect was dependent on the intensity of NIR light (n=3; error, SE.).

(B) No therapeutic effect was observed in weakly positive or negative cells of EGFR or HER2 expression.

Supplemental Figure S3: Dual- targeted NIR-PIT in vitro

A TE4

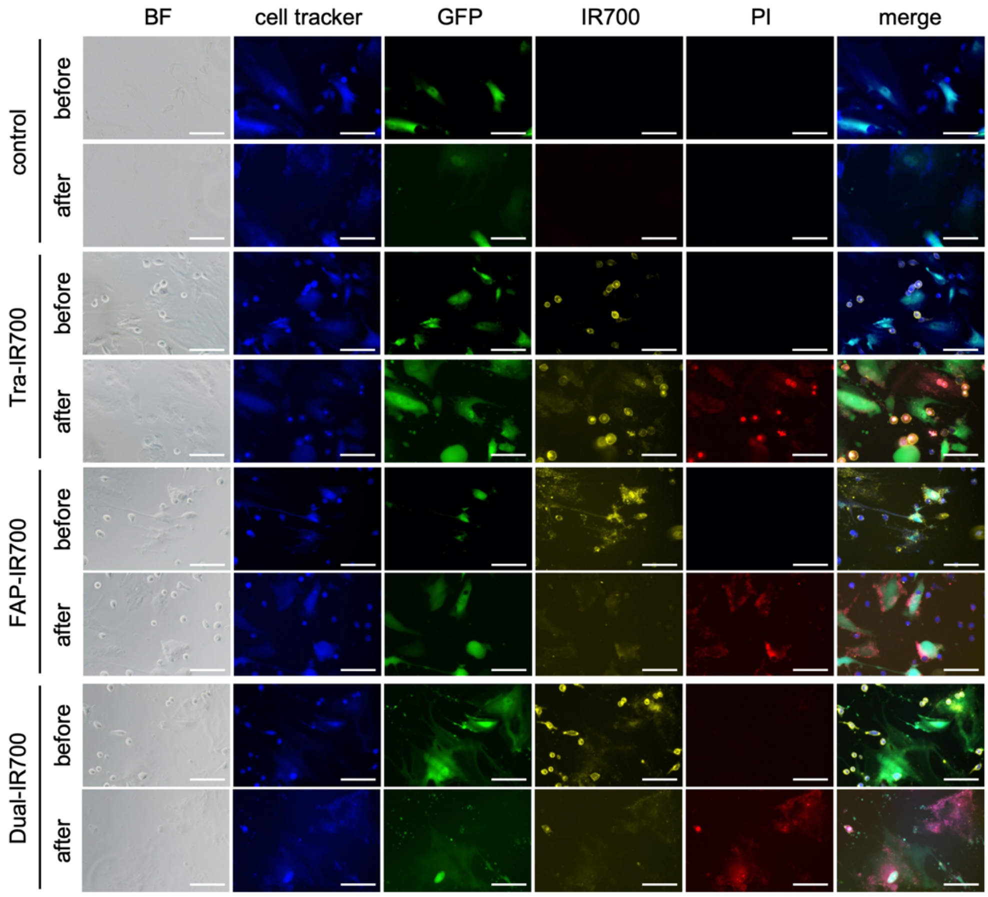

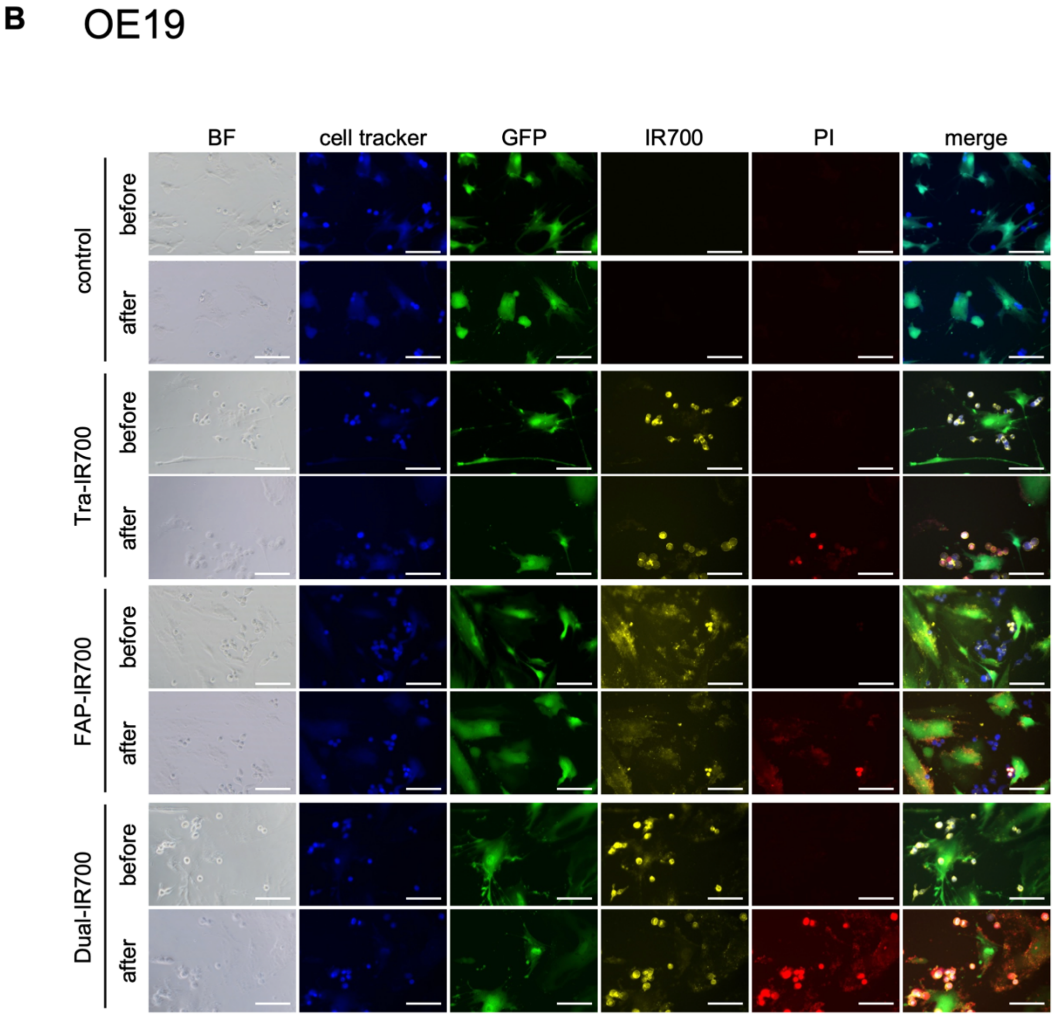

Supplemental Figure S3. Dual- targeted NIR-PIT in vitro

Immunofluorescent microscopy presenting NIR-PIT under co-culture of TE4 and FEF3 (A), OE19 and FEF3 (B). In the bright field, TE4 and OE19 cells are recognized as round cells,

morphologically distinct from spindle-shaped FEF3 cells. Cultures were stained for cancer cells (cell tracker; blue), GFP-FEF3 (GFP; green), mAb-IR700 (IR700; yellow), and dead cells (PI; red). PI was not observed in cells not targeted or surrounding cells in mono- (Cancer cell-, CAF-) Targeted NIR-PIT under co-cultivation. Dual- Targeted NIR-PIT showed an additive effect, confirming that cancer cells and CAFs could be treated simultaneously with a single NIR light irradiation. Scale bars: 50 μm.

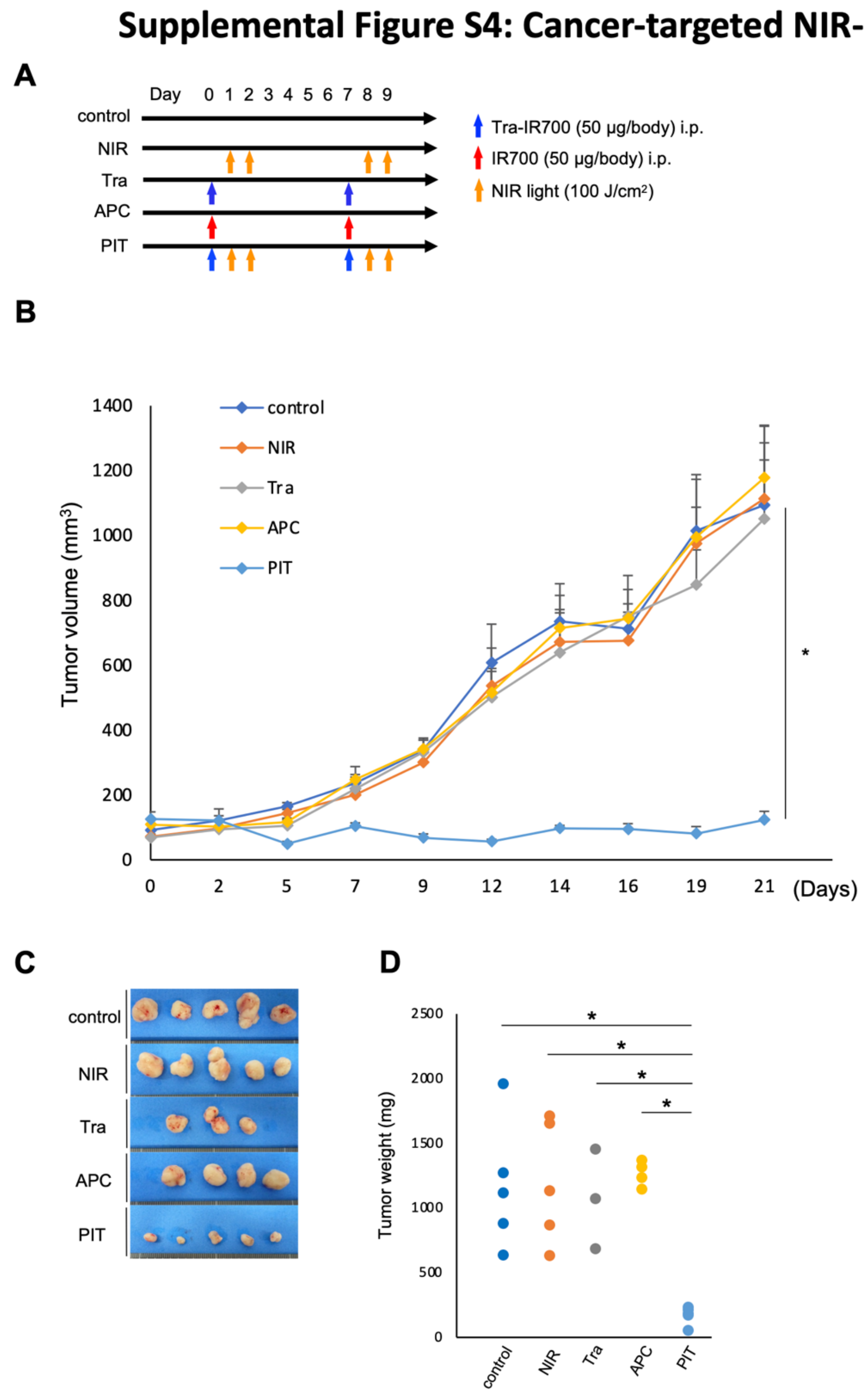

Supplemental Figure S4. Cancer cell-targeted NIR-PIT in vivo

TE4 cells (3.0×10^6 cells) were suspended in PBS (50 µL) and Basement Membrane Matrix (100 µL) (BD Biosciences) and were injected subcutaneously into the right flank of

(BALB/c-*nu/nu* mice. When the tumor reached $100mm^3$ after injection, the mice randomized into four groups; (a) no treatment group(control); treatment groups by (b) irradiation with NIR light at 50 J/cm^2 (NIR only); (c) intraperitoneally (i.p.) injection with 50 µg/body of Trastuzumab (Tra only); (d) i.p. injection with 50 µg/body of Tra- IR700 (APC only); (e) i.p. injection with 50 µg/body of Tra-IR700 plus irradiation with NIR light at 50 J/cm^2 (PIT).

(A) Treatment protocol

(B) Tumor growth of subcutaneous tumors inoculated in BALB/c-*nu/nu* mice. The tumors were dramatically suppressed in PIT group compared with others (mean ± SEM. *$P < 0.05$, Tukey's test with ANOVA).

(C, D) Evaluation of tumor weight is shown for each group (*$P < 0.05$, Tukey's test with ANOVA).

Supplemental Figure S5: OS in esophageal SCC patients

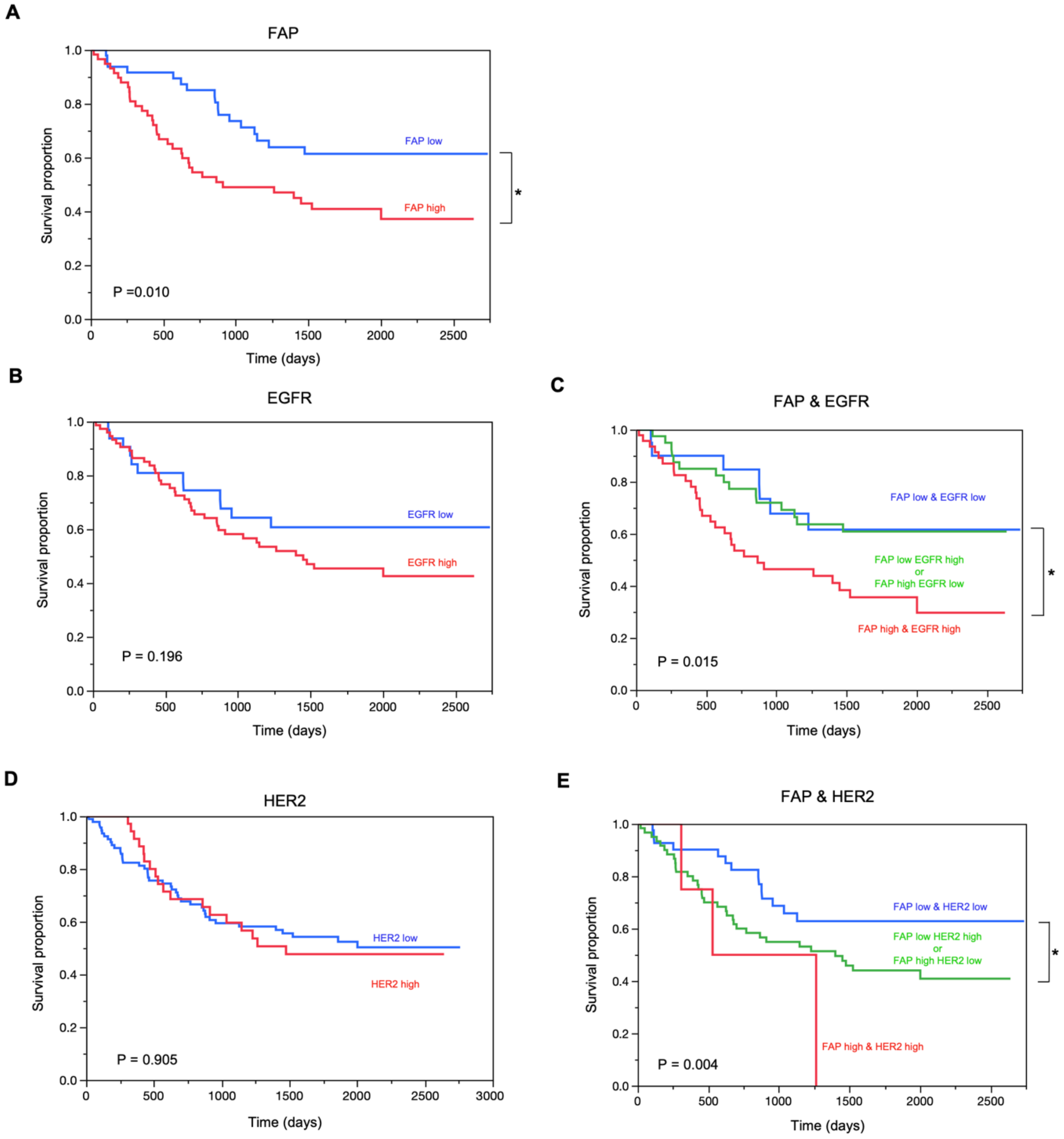

Supplemental Figure S5. Clinical outcome in 109 cases of esophageal squamous cell carcinoma

(A) Survival analysis showed that FAP high patients had significantly worse OS than those with low FAP (*P* =0.010, Log-Rank)

(B) No difference was observed between patients with high EGFR and those with low EGFR in the analysis for OS (*P* = 0.196, Log-Rank).

(C) Survival curve of three groups divided by combination of EGFR and FAP scores (double negative, single positive, double positive), double positive group had worse survival than single positive group and double negative group (*P* = 0.012: double positive vs single negative, *P* =0.041: double positive vs double negative, log-rank test; *, *P* <0.05)

(D) No difference was observed between patients with high HER2 and those with low HER2 in the analysis for OS (*P* = 0.905, Log-Rank).

(E) Survival curve of three groups divided by combination of HER2 and FAP scores (double negative, single positive, double positive), single positive group and double positive group had worse survival than double negative group (*P* = 0.047: single positive vs double negative, *P* =0.054: double positive vs double negative, log-rank test; *, *P* <0.05)

Supplemental Table S1: Clinicopathological characteristics of the study patients

Variables	Total	FAP			EGFR			HER2		
		Low (≤3)	High (>3)	*P* value	Low (≤1)	High (>1)	*P* value	Low (≤1)	High (>1)	*P* value
No. of patients	132	67	65		51	81		115	17	
Age (median)				0.905§			0.223§			0.672§
Median (IQR)	67 (61-72)	66 (61-72)	67 (61-73)		69 (63-73)	65 (61-72)		67 (61-72)	67 (61-72)	
Sex				0.816†			0.338†			0.416†
Male	110	55	55		45	65		97	13	
Female	22	12	10		6	16		18	4	
Tumor depth				<0.001†*			<0.001†*			0.940†
Tx, T1	61	49	12		17	54		53	8	
T2-4	71	18	53		34	27		62	9	
Lymph node				<0.001†*			0.814†			0.953†
Negative	63	43	20		25	38		55	8	
Positive	69	24	45		26	43		60	9	
Histological type				0.061‡			<0.001‡*			0.633‡
SCC										
well	24	10	14		5	19		21	3	
moderate	63	28	35		22	41		56	7	
poor	22	11	11		6	16		20	2	
Adenocarcinoma	7	6	1		6	1		6	1	
Other	16	12	4		12	4		12	4	
Neoadjuvant therapy				0.178‡			0.074‡			0.330‡
none	101	50	51		42	59		86	15	
chemotherapy	19	8	11		3	16		17	2	
chemoradiotherapy	12	9	3		6	6		12	0	
FAP score							<0.001‡*			0.350‡
0	28				23	5		24	4	
1	6				4	2		6	0	
2	12				4	8		11	1	
3	21				4	17		16	5	
4	26				9	17		21	5	
5	17				5	12		16	1	
6	17				2	15		17	0	
7	5				0	5		4	1	
EGFR score				<0.001‡*						0.573‡
0	30	25	5					28	2	
1	21	10	11					18	3	
2	39	19	20					32	7	
3	42	13	29					37	5	
HER2 score				0.407‡			0.136‡			
0	95	47	48		39	56				
1	20	10	10		7	13				
2	13	9	4		2	11				
3	4	1	3		3	1				

§Student's t-test, †Fisher's exact test; ‡Peason's chi-square test, *Statistical significance at P-value <0.05. IQR, interquartile range; SCC, squamous cell carcinoma; FAP, fibroblast activation protein; EGFR, epidermal growth factor receptor; HER2, human epidermal growth factor 2

Supplemental Table S2: Univariate analysis of clinicopathological features for OS and DFS in esophageal cancer patients

		OS			DFS		
**Variable**	**Unfavorable/Favorable**	**HR**	**95%CI**	***P* value**	**HR**	**95%CI**	***P* value**
Age (years)	>67/≤67	0.92	0.55-1.53	0.755	0.89	0.544-1.45	0.638
Sex	Female/Male	2.65	1.16-7.60	0.017*	2.75	1.21-7.87	0.013*
Tumor depth	Tis, T1/ T2-T4	3.29	1.89-6.01	<0.001*	3.71	2.16-6.66	<0.001*
Lymph node	negative/ positive	3.48	2.00-6.37	<0.001*	3.44	2.02-6.10	<0.001*
FAP score	Low (≤3)/ high (>3)	2.37	1.41-4.08	0.001*	2.37	1.44-4.00	<0.001*
EGFR score	Low (≤1)/ high (>1)	1.64	0.96-2.93	0.069	1.94	1.14-3.43	0.013*
HER2 score	Low (≤1)/ high (>1)	1.37	0.65-2.59	0.380	1.28	0.61-2.41	0.486

Cox proportional hazards regression. *Statistical significance at P-value <0.05. OS, overall survival; DFS, disease free survival; HR, hazard ratio; CI, confidence interval; SCC, squamous cell carcinoma; FAP, fibroblast activation protein; EGFR, epidermal growth factor receptor; HER2, human epidermal growth factor 2

Supplemental Table S3: Clinicopathological characteristics of esophageal squamous cell carcinoma patients

		FAP			EGFR			HER2		
Variables	Total	Low (≤3)	High (>3)	*P* value	Low (≤1)	High (>1)	*P* value	Low (≤1)	High (>1)	*P* value
No. of patients	109	67	65		51	81		115	17	
Age (median)				0.732§			0.461§			0.247§
Median (IQR)	66 (61-72)	65 (61-71)	67 (61-72)		67 (62-72)	65 (61-72)		66 (61-71)	68 (62-74)	
Sex				0.816†			0.130†			0.124†
Male	90	40	50		30	60		82	8	
Female	19	9	10		3	16		15	4	
Tumor depth				<0.001†*			<0.001†*			0.323†
Tx, T1	49	37	12		23	26		42	7	
T2-4	60	12	48		10	50		55	5	
Lymph node				<0.001†*			0.691†			0.920†
Negative	53	33	20		17	36		47	6	
Positive	56	16	40		16	40		50	6	
Histological type				0.845‡			0.417‡			0.933‡
SCC										
well	24	10	14		5	19		21	3	
moderate	63	28	35		22	41		56	7	
poor	22	11	11		6	16		20	2	
Adenocarcinoma	0	0	0		0	0		0	0	
Other	0	0	0		0	0		0	0	
Neoadjuvant therapy				0.113‡			0.119‡			0.540‡
none	82	35	47		27	55		72	10	
chemotherapy	18	7	11		2	16		16	2	
chemoradiotherapy	9	7	2		4	5		9	0	
FAP score							<0.001‡*			0.655‡
0	16				12	4		13	3	
1	5				3	2		2	0	
2	19				2	7		8	1	
3	19				4	15		16	3	
4	22				6	16		19	3	
5	17				5	12		16	1	
6	16				1	15		16	0	
7	5				0	5		4	1	
EGFR score				0.024‡*						0.888‡
0	18	13	5					16	2	
1	15	8	7					14	1	
2	36	16	20					31	5	
3	40	12	28					36	4	
HER2 score				0.753‡			0.827‡			
0	78	33	45		24	54				
1	19	9	10		6	13				
2	10	6	4		2	8				
3	2	1	1		1	1				

§Student's t-test, †Fisher's exact test; ‡Peason's chi-square test, *Statistical significance at P-value <0.05. IQR, interquartile range; SCC, squamous cell carcinoma; FAP, fibroblast activation protein; EGFR, epidermal growth factor receptor; HER2, human epidermal growth factor 2

Supplemental Table S4: Univariate analysis of clinicopathological features for OS and DFS in esophageal squamous cell carcinoma patients

		OS			DFS		
**Variable**	**Unfavorable/Favorable**	**HR**	**95%CI**	***P* value**	**HR**	**95%CI**	***P* value**
Age (years)	>67/≤67	0.88	0.50-1.53	0.650	0.81	0.47-1.39	0.453
Sex	Female/Male	3.05	1.24-10.14	0.01*	3.14	1.28-10.40	0.010*
Tumor depth	Tis, T1/ T2-T4	3.42	1.87-6.69	<0.001*	3.82	2.12-7.30	<0.001*
Lymph node	negative/ positive	3.52	1.94-6.76	<0.001*	3.54	2.01-6.55	<0.001*
FAP score	Low (≤3)/ high (>3)	2.11	1.20-3.88	0.010*	2.23	1.29-4.02	0.004*
EGFR score	Low (≤1)/ high (>1)	1.53	0.82-3.05	0.184	1.83	0.99-3.62	0.052
HER2 score	Low (≤1)/ high (>1)	0.97	0.37-2.10	0.940	0.89	0.34-1.91	0.784

Cox proportional hazards regression. *Statistical significance at P-value <0.05. OS, overall survival; DFS, disease free survival; HR, hazard ratio; CI, confidence interval; SCC, squamous cell carcinoma; FAP, fibroblast activation protein; EGFR, epidermal growth factor receptor; HER2, human epidermal growth factor 2

Supplementary Information of multiple exposure images of original blots with molecular size markings.

Figure 2A_1-1

EGFR (HSC-2, T.Tn, TE1, TE4, TE6, TE8, TE10)

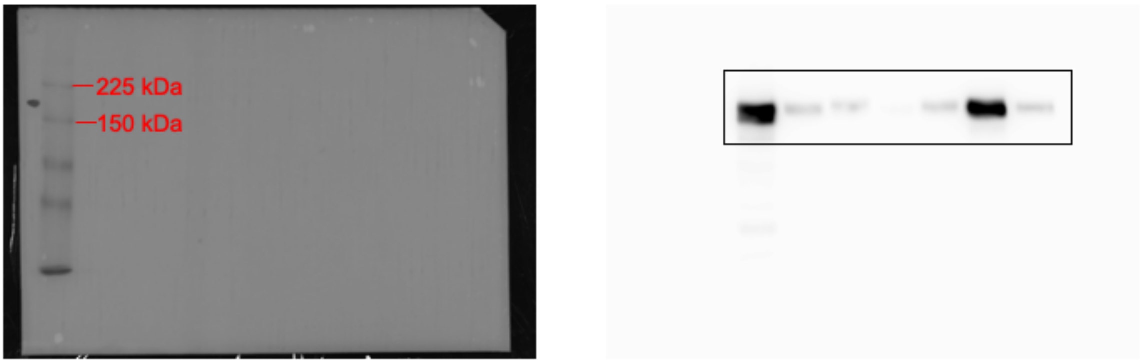

Figure 2A_1-2

EGFR (HSC-2, T.Tn, TE1, TE4, TE6, TE8, TE10)

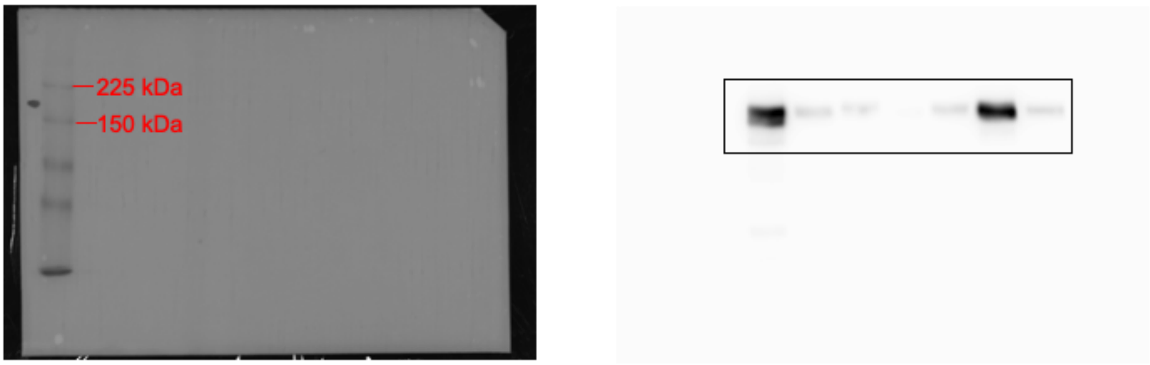

Figure 2A_1-3

EGFR (HSC-2, T.Tn, TE1, TE4, TE6, TE8, TE10)

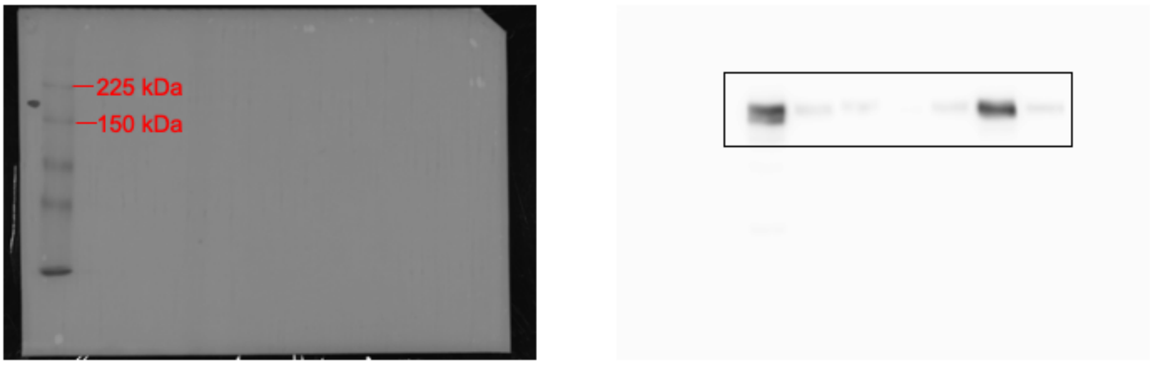

Figure 2A_1-4

EGFR (HSC-2, T.Tn, TE1, TE4, TE6, TE8, TE10)

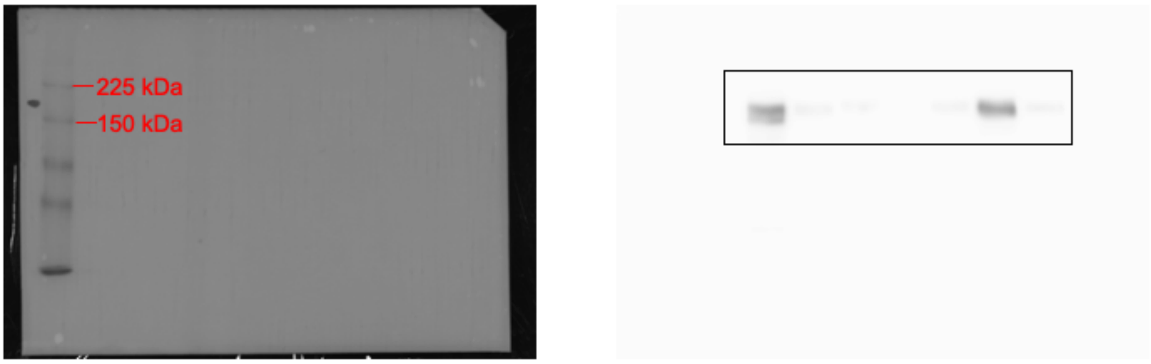

Figure 2A_2-1

B-actin (HSC-2, T.Tn, TE1, TE4, TE6, TE8, TE10)

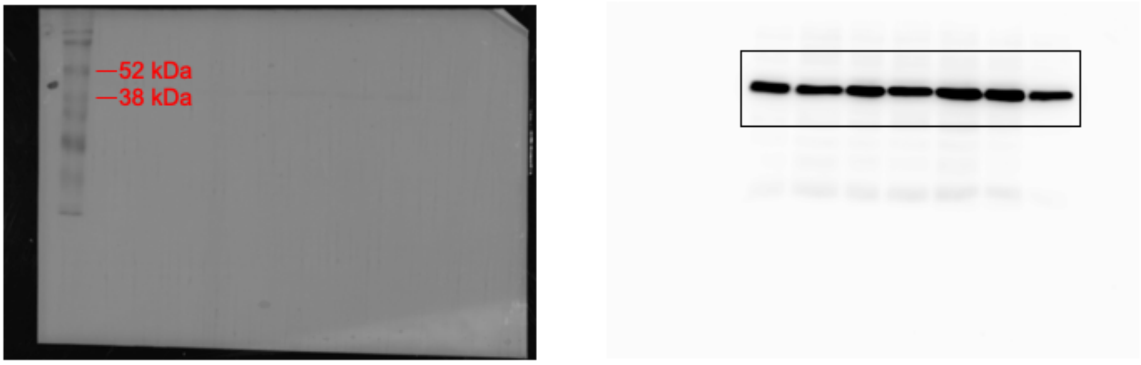

Figure 2A_2-2

B-actin (HSC-2, T.Tn, TE1, TE4, TE6, TE8, TE10)

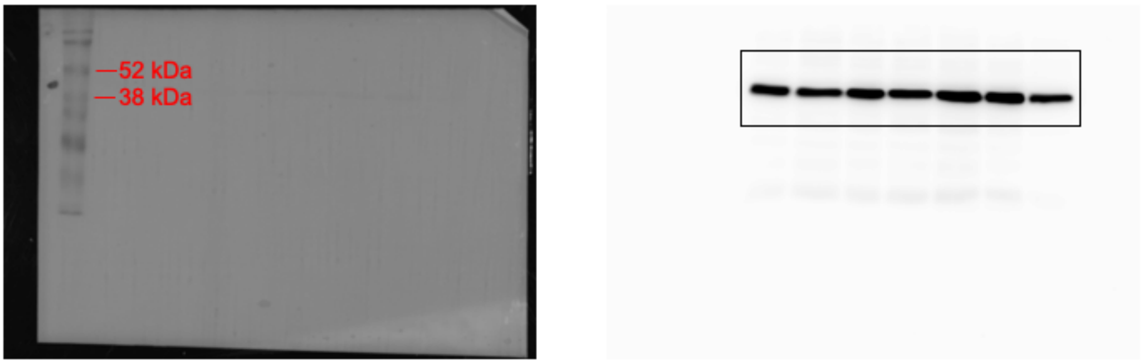

Figure 2A_2-3

B-actin (HSC-2, T.Tn, TE1, TE4, TE6, TE8, TE10)

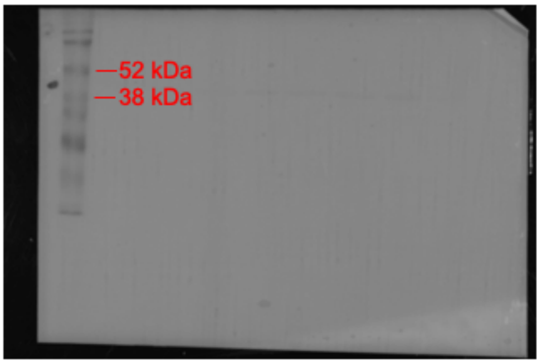

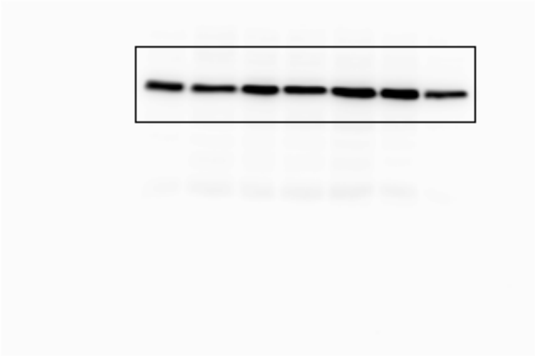

Figure 2A_2-4

B-actin (HSC-2, T.Tn, TE1, TE4, TE6, TE8, TE10)

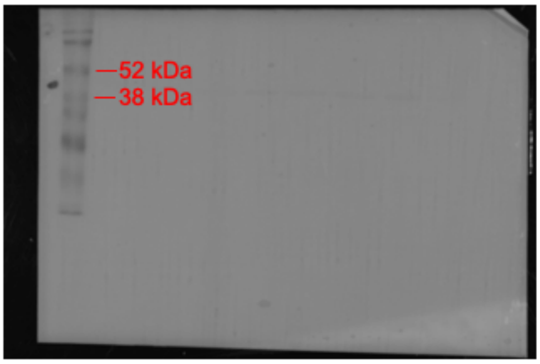

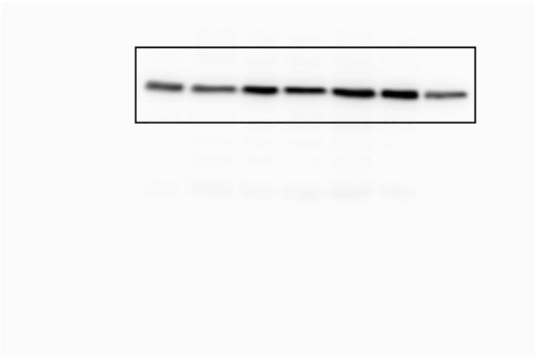

Figure 2A_2-5

B-actin (HSC-2, T.Tn, TE1, TE4, TE6, TE8, TE10)

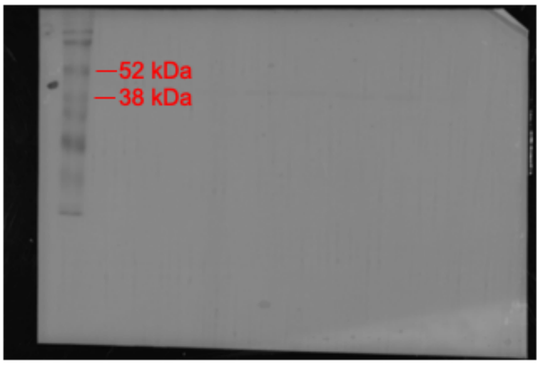

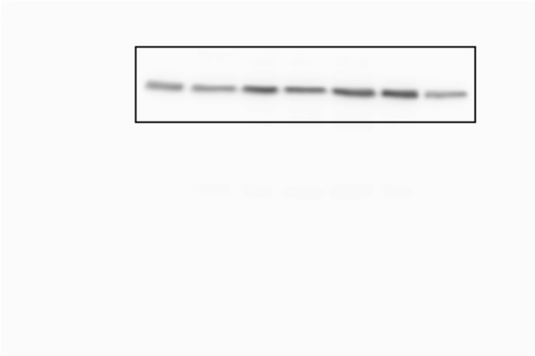

Figure 2A_3-1

EGFR (TE13, TE14, TE15, OE19, OE33)

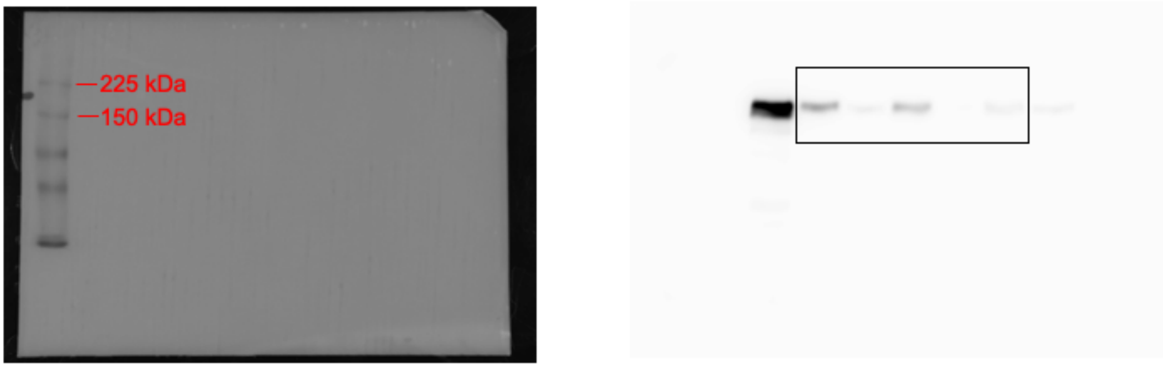

Figure 2A_3-2

EGFR (TE13, TE14, TE15, OE19, OE33)

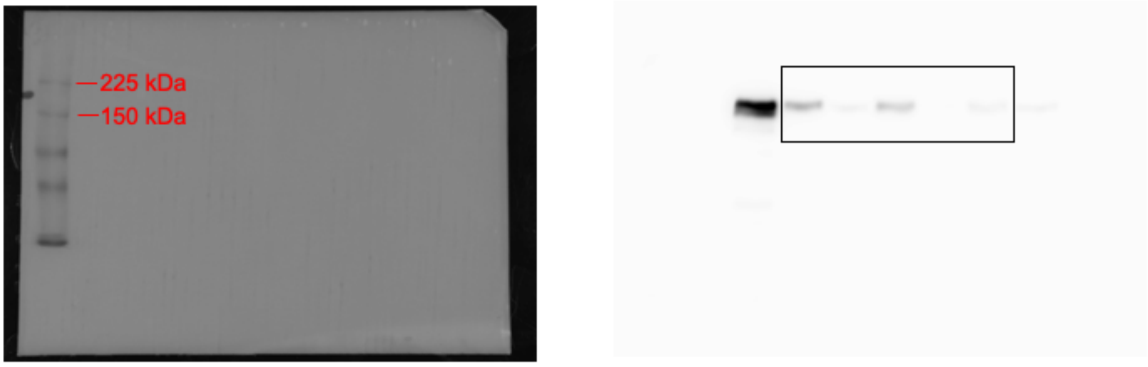

Figure 2A_3-3

EGFR (TE13, TE14, TE15, OE19, OE33)

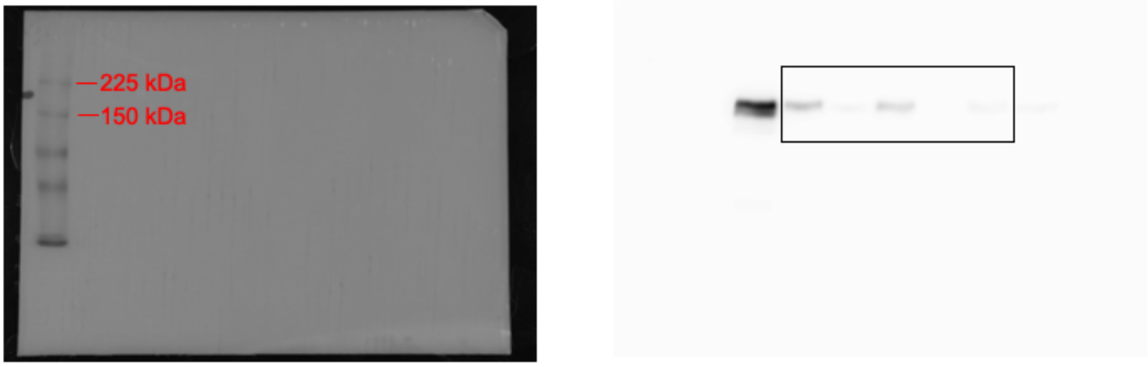

Figure 2A_3-4

EGFR (TE13, TE14, TE15, OE19, OE33)

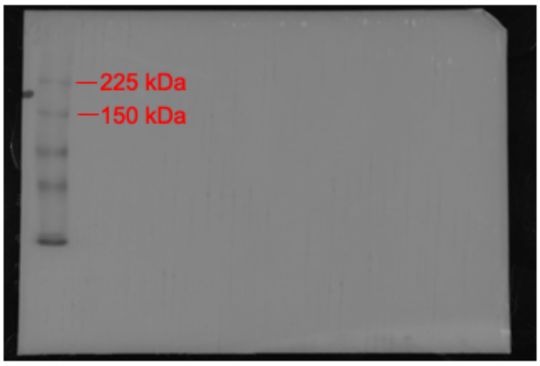

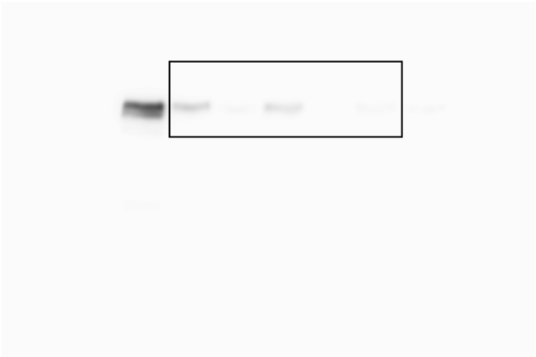

Figure 2A_3-5

EGFR (TE13, TE14, TE15, OE19, OE33)

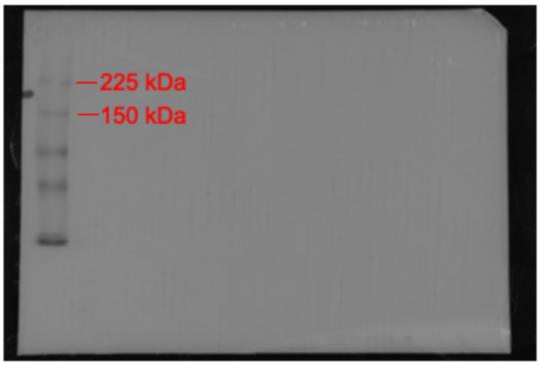

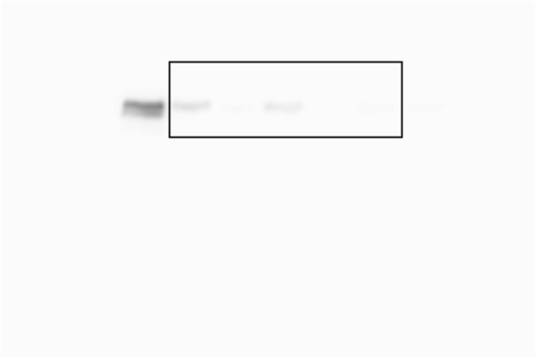

Figure 2A_3-6

EGFR (TE13, TE14, TE15, OE19, OE33)

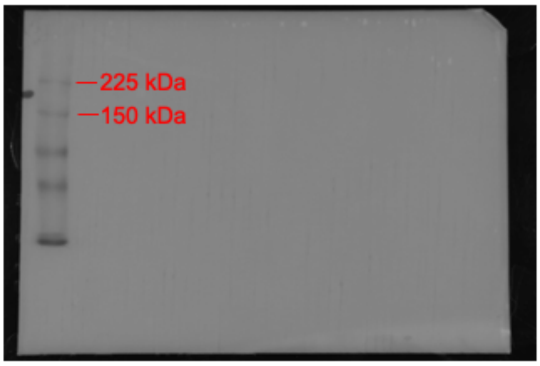

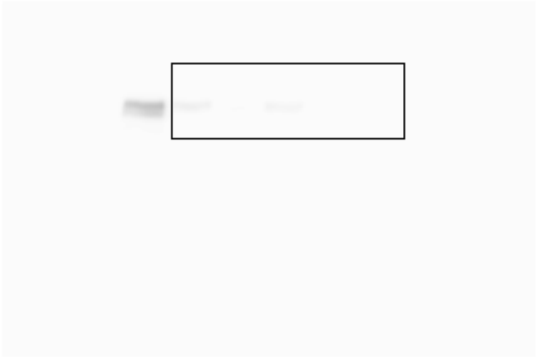

Figure 2A_4-1

B-actin (TE13, TE14, TE15, OE19, OE33)

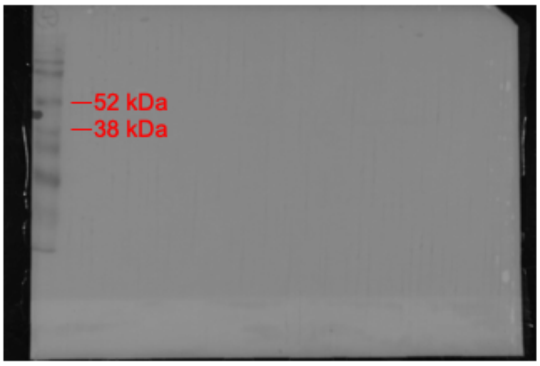

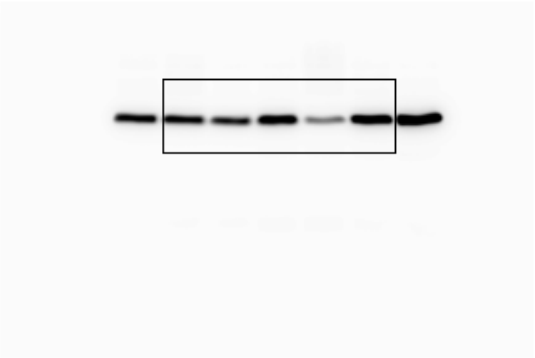

Figure 2A_4-2

B-actin (TE13, TE14, TE15, OE19, OE33)

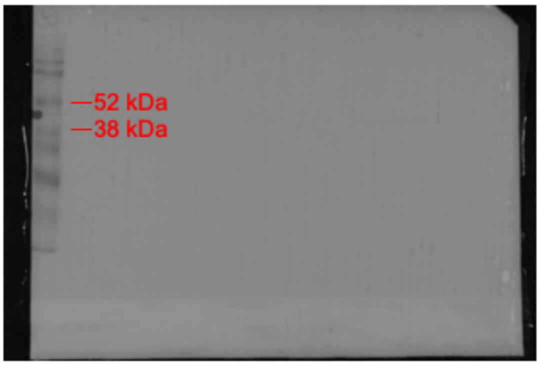

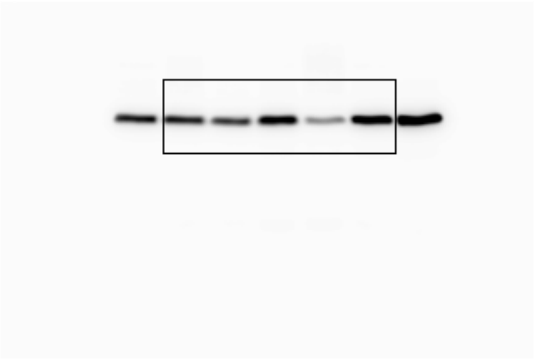

Figure 2A_4-3

B-actin (TE13, TE14, TE15, OE19, OE33)

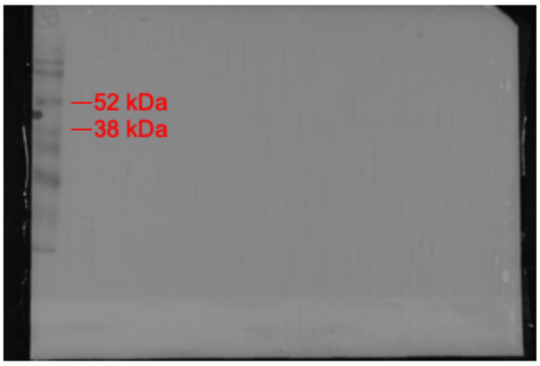

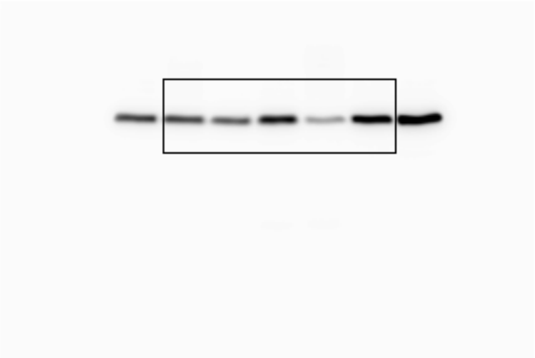

Figure 2A_4-4

B-actin (TE13, TE14, TE15, OE19, OE33)

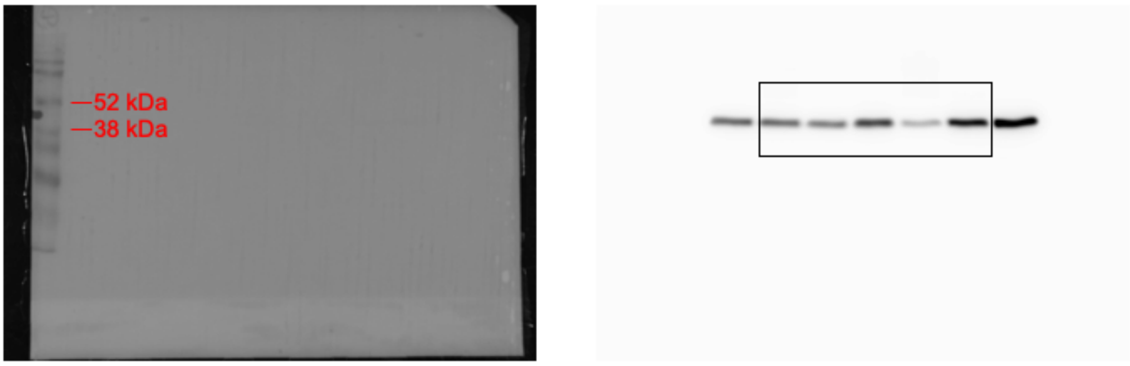

Figure 2A_4-5

B-actin (TE13, TE14, TE15, OE19, OE33)

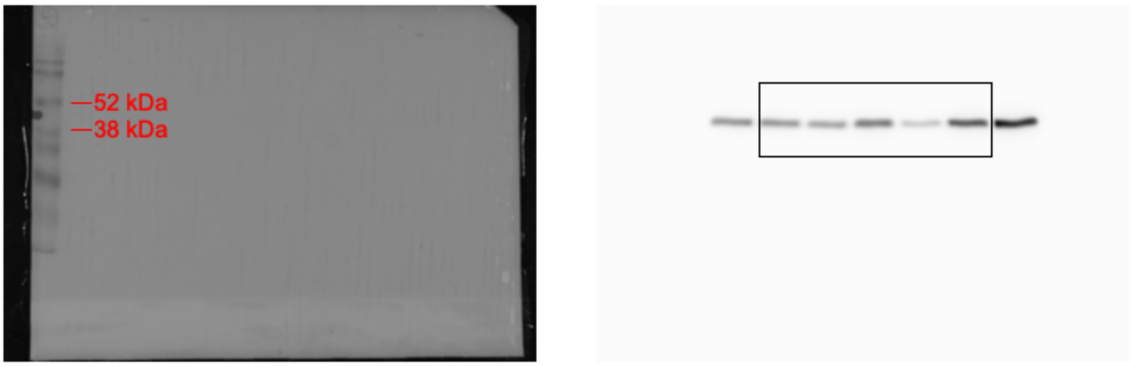

Figure 2A_4-6

B-actin (TE13, TE14, TE15, OE19, OE33)

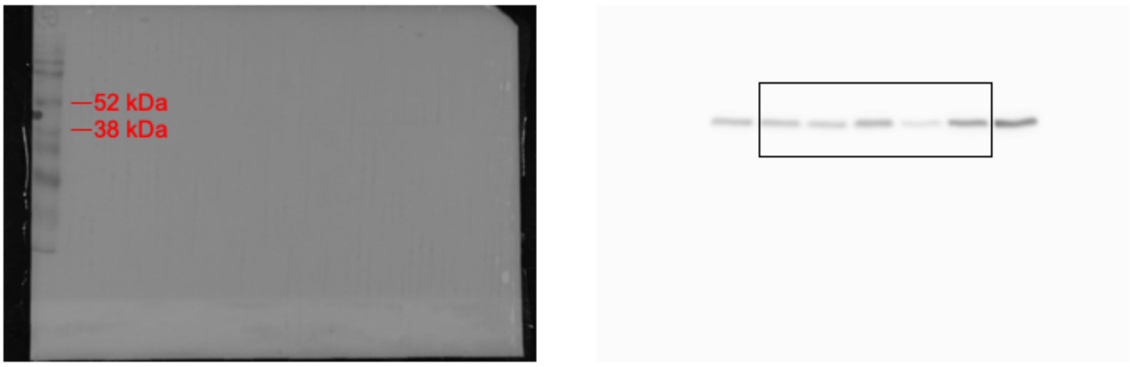

Figure 2B_5-1

HER2 (SR-BK-3, T.Tn, TE1, TE4, TE6)

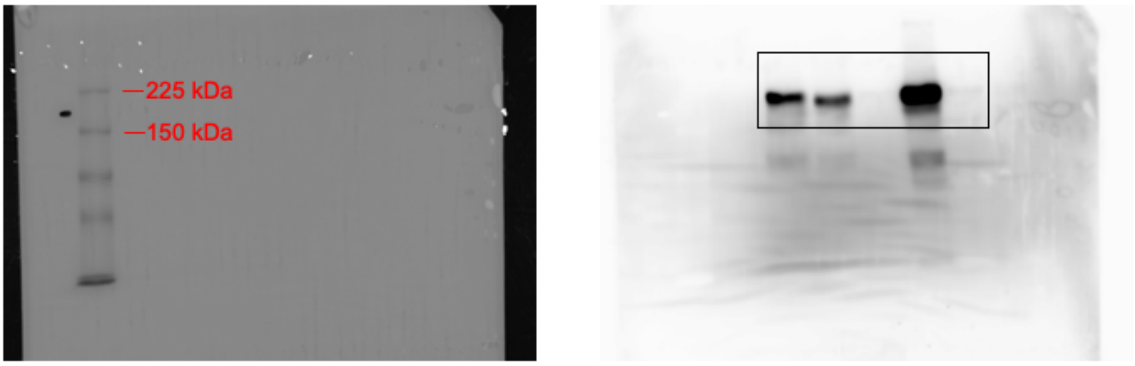

Figure 2B_5-2

HER2 (SR-BK-3, T.Tn, TE1, TE4, TE6)

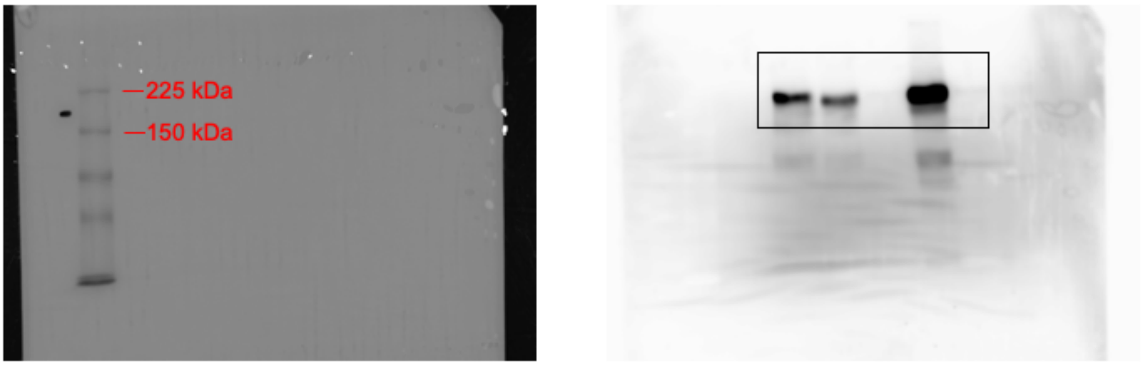

Figure 2B_5-3

HER2 (SR-BK-3, T.Tn, TE1, TE4, TE6)

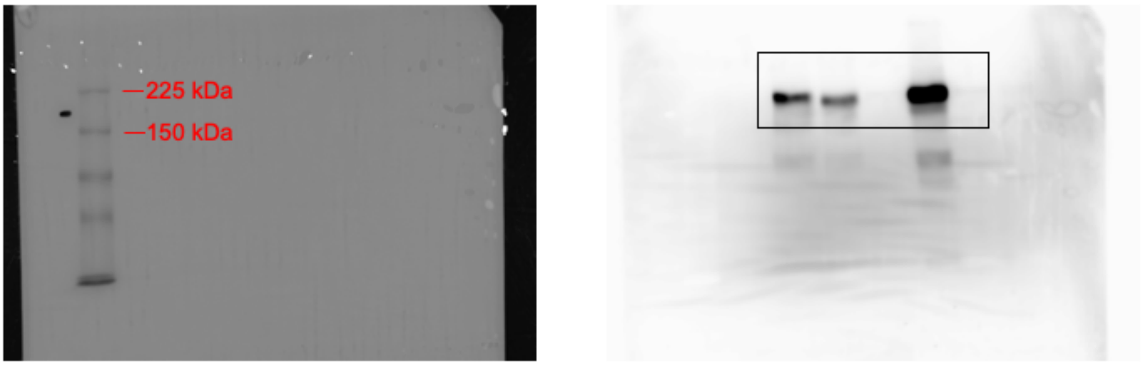

Figure 2B_5-4

HER2 (SR-BK-3, T.Tn, TE1, TE4, TE6)

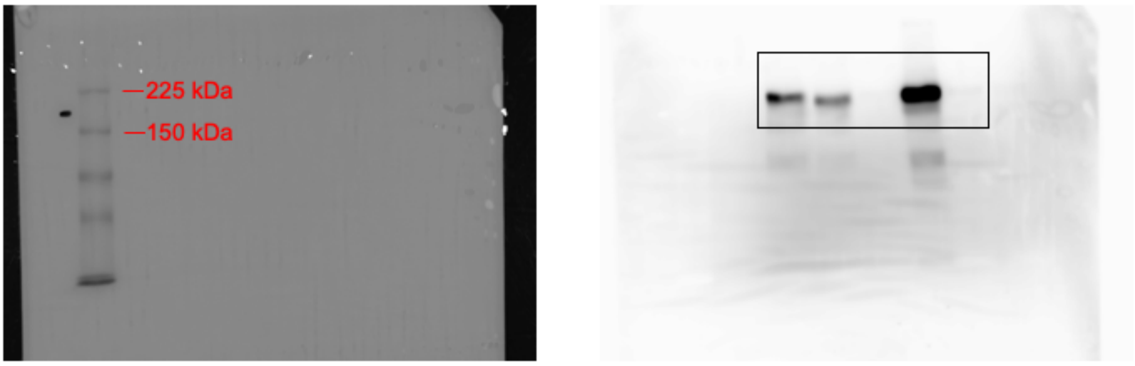

Figure 2B_5-5

HER2 (SR-BK-3, T.Tn, TE1, TE4, TE6)

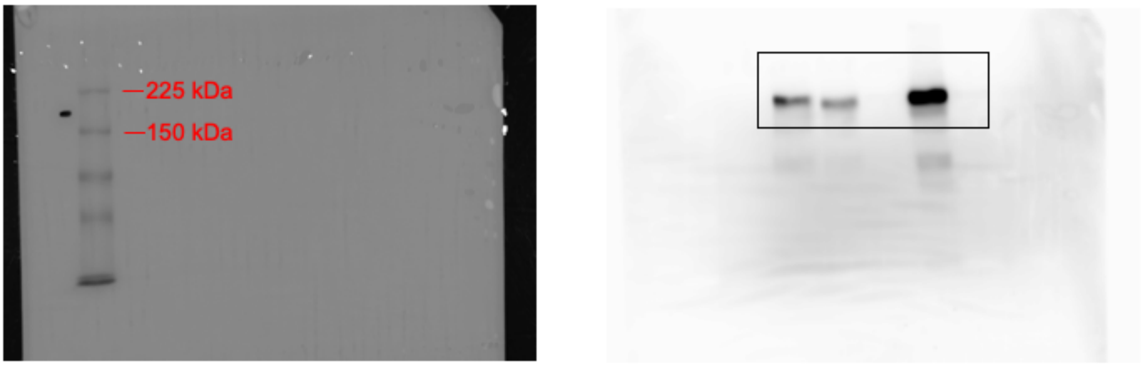

Figure 2B_5-6

HER2 (SR-BK-3, T.Tn, TE1, TE4, TE6)

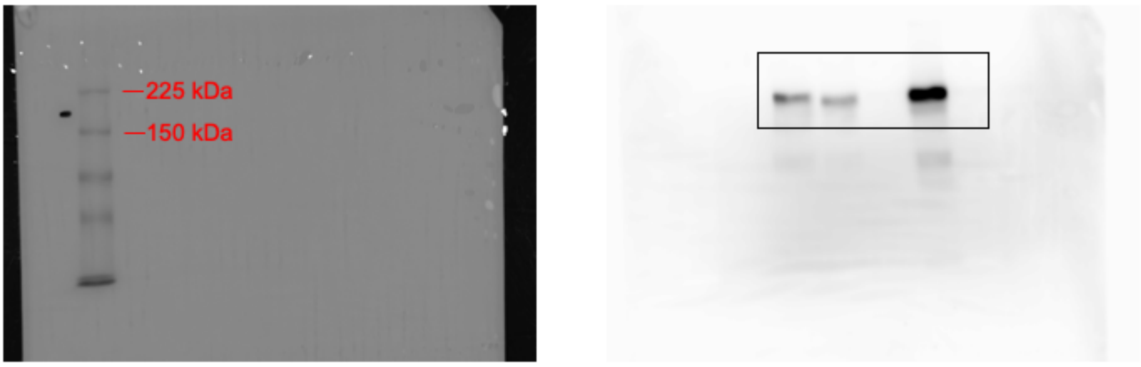

Figure 2B_5-7

HER2 (SR-BK-3, T.Tn, TE1, TE4, TE6)

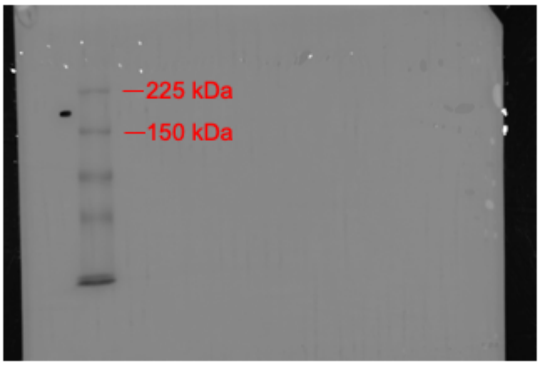

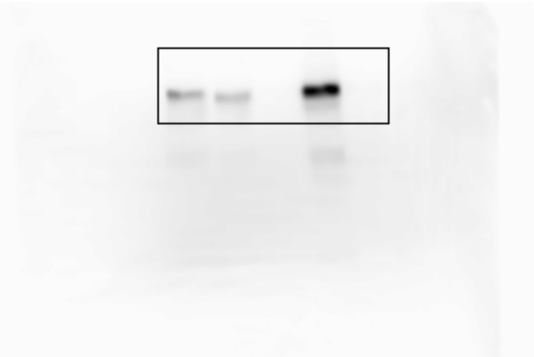

Figure 2B_5-8

HER2 (SR-BK-3, T.Tn, TE1, TE4, TE6)

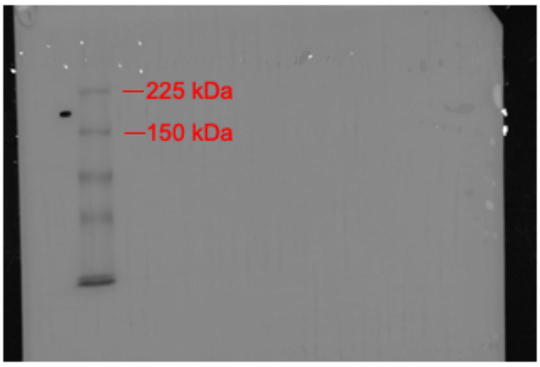

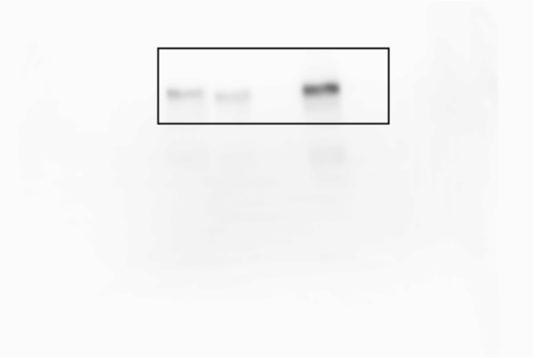

Figure 2B_6-1

B-actin (SR-BK-3, T.Tn, TE1, TE4, TE6)

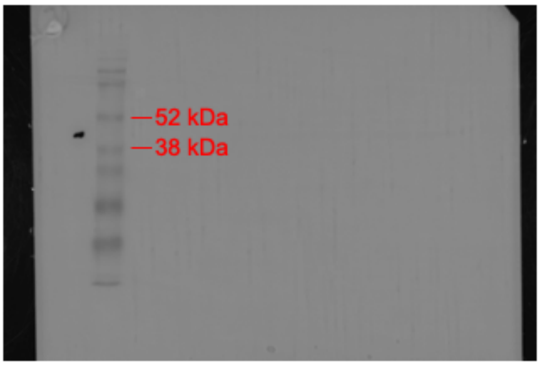

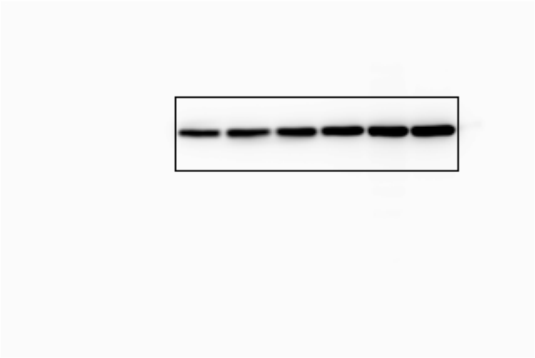

Figure 2B_6-2

B-actin (SR-BK-3, T.Tn, TE1, TE4, TE6)

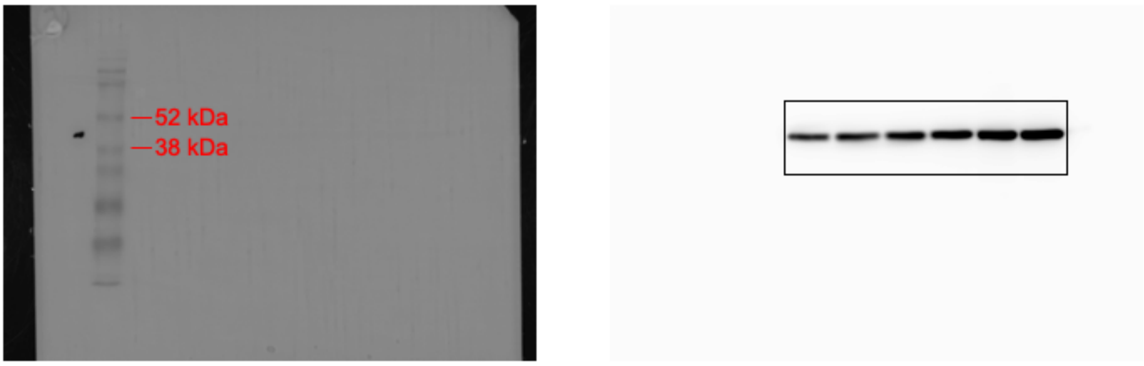

Figure 2B_6-3

B-actin (SR-BK-3, T.Tn, TE1, TE4, TE6)

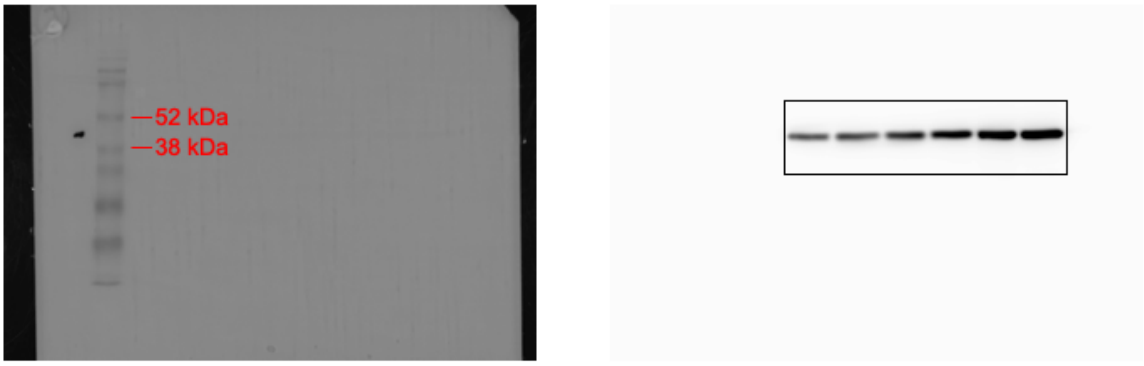

Figure 2B_6-4

B-actin (SR-BK-3, T.Tn, TE1, TE4, TE6)

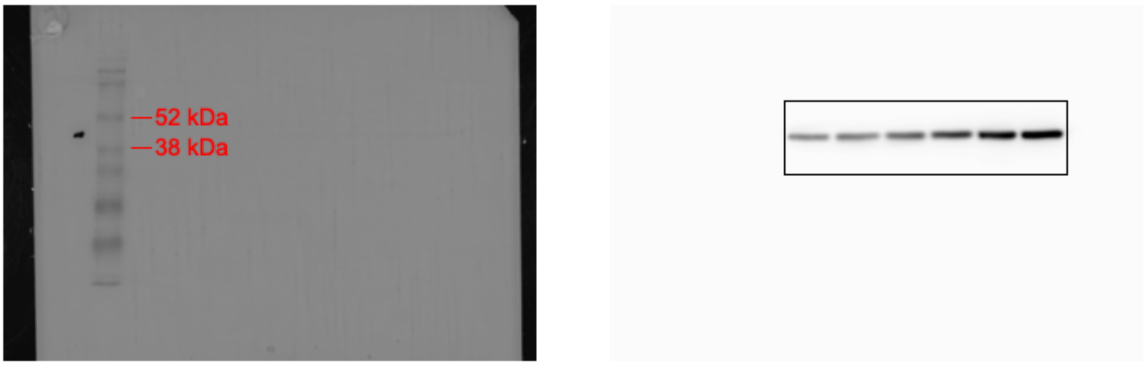

Figure 2B_6-5

B-actin (SR-BK-3, T.Tn, TE1, TE4, TE6)

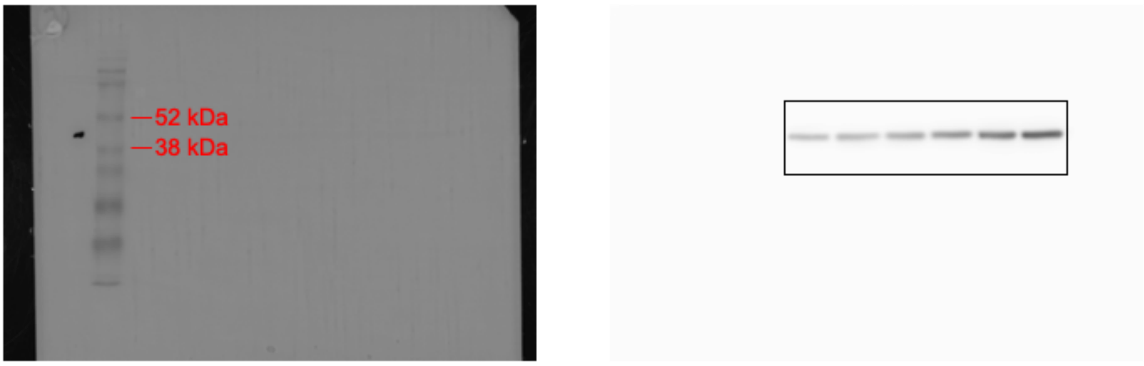

Figure 2B_7-1

HER2 (TE8, TE10, TE13, TE14)

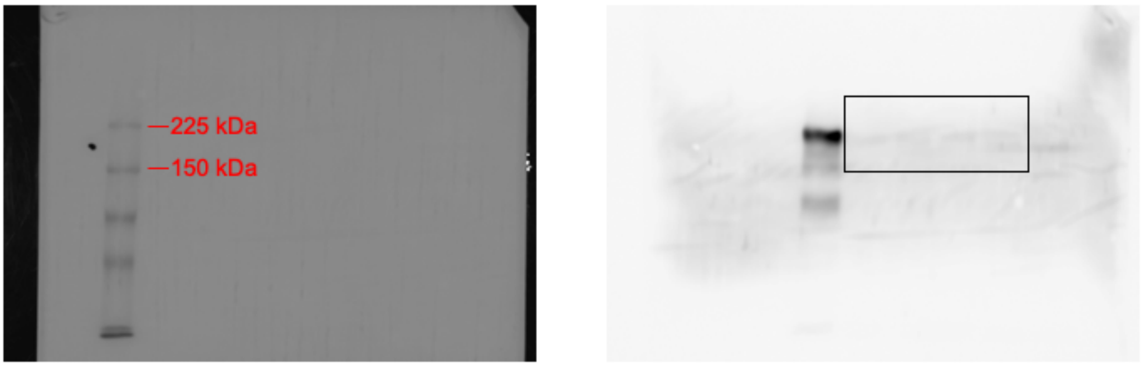

Figure 2B_7-2

HER2 (TE8, TE10, TE13, TE14)

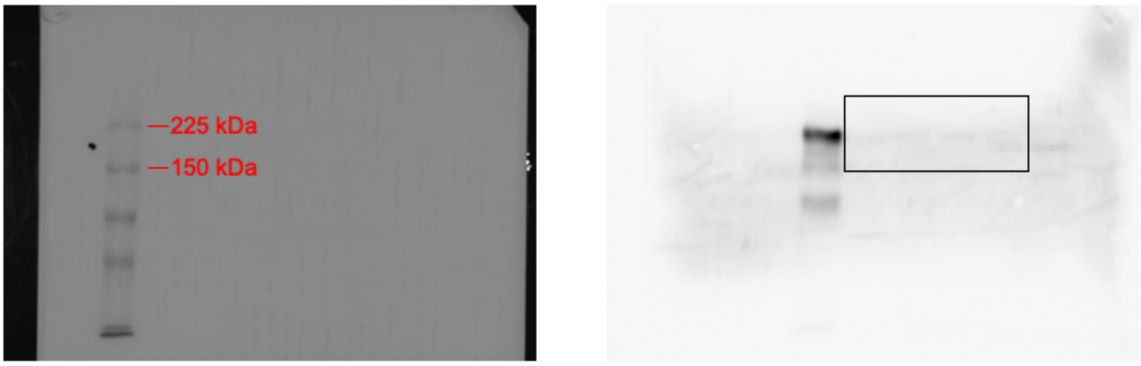

Figure 2B_7-3

HER2 (TE8, TE10, TE13, TE14)

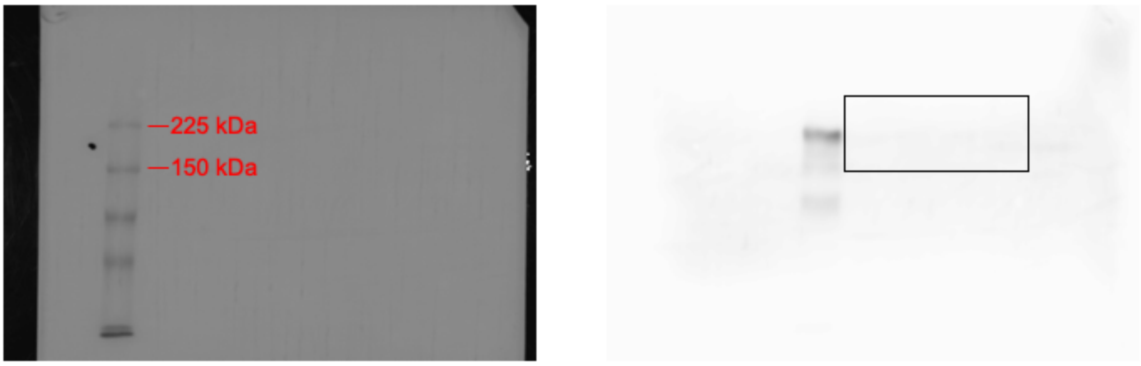

Figure 2B_7-4

HER2 (TE8, TE10, TE13, TE14)

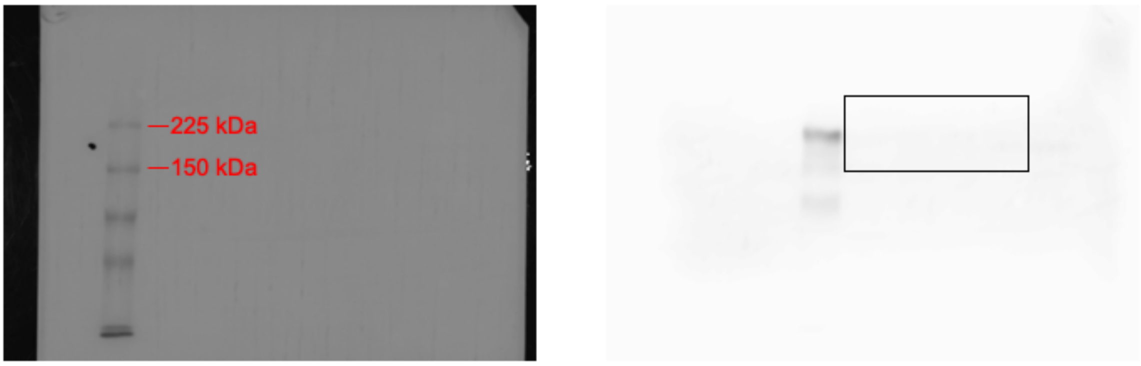

Figure 2B_7-5

HER2 (TE8, TE10, TE13, TE14)

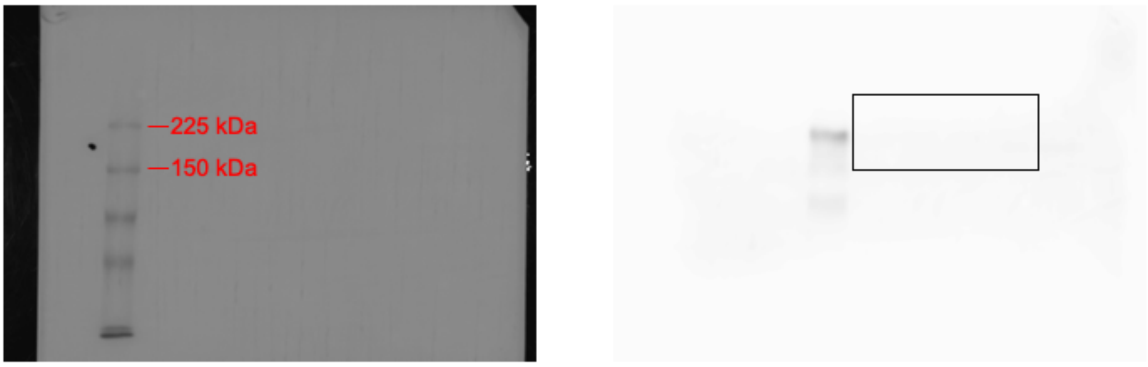

Figure 2B_7-6

HER2 (TE8, TE10, TE13, TE14)

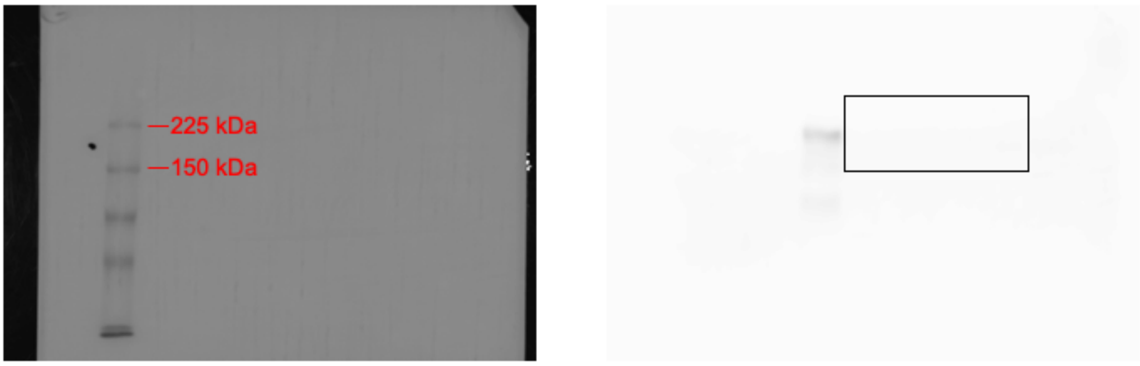

Figure 2B_7-7

HER2 (TE8, TE10, TE13, TE14)

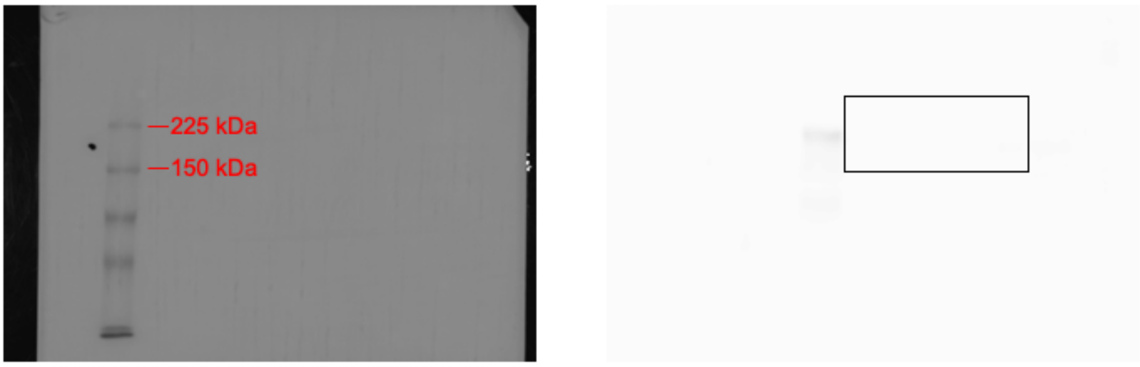

Figure 2B_8-1

B-actin (TE8, TE10, TE13, TE14)

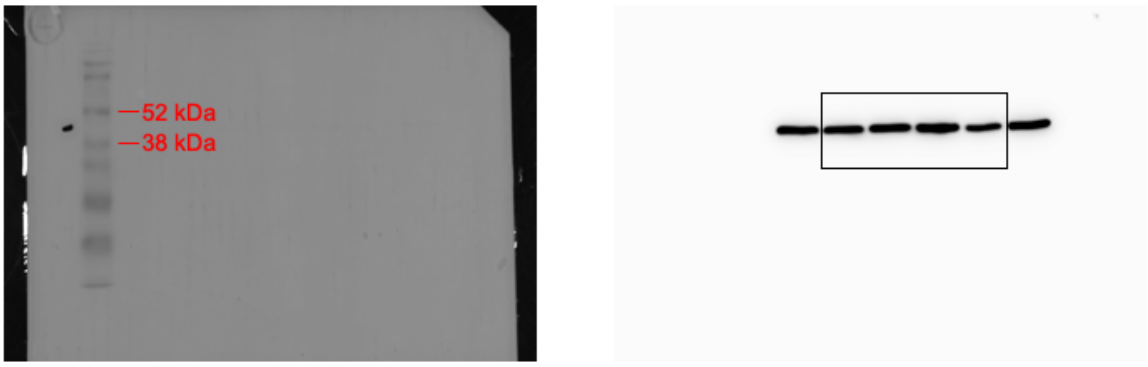

Figure 2B_8-2

B-actin (TE8, TE10, TE13, TE14)

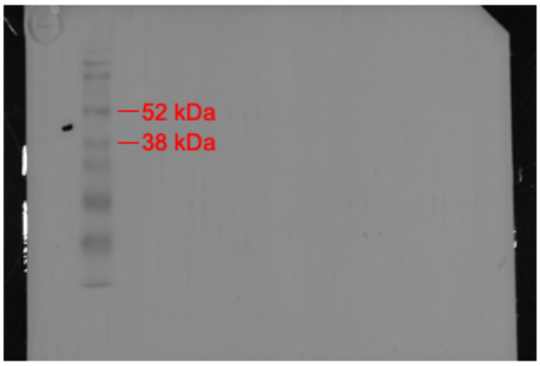

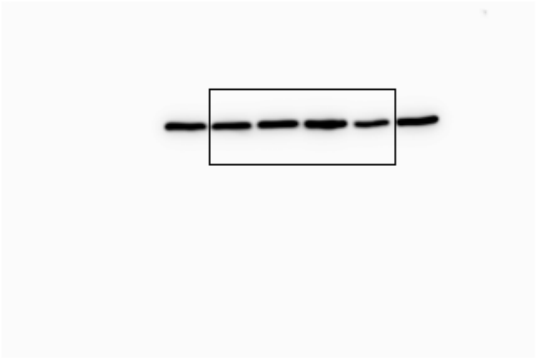

Figure 2B_8-3

B-actin (TE8, TE10, TE13, TE14)

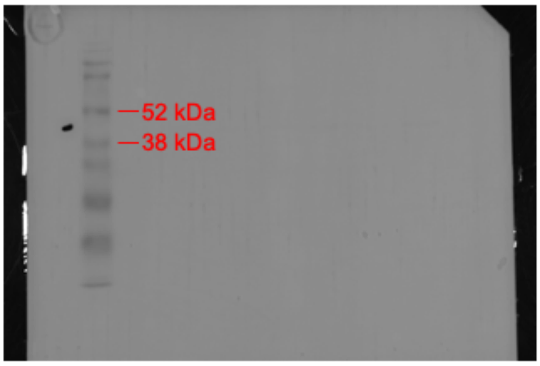

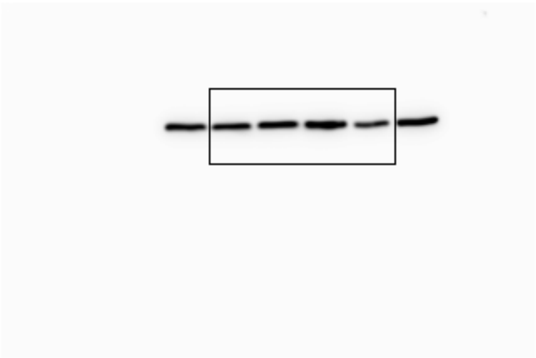

Figure 2B_8-4

B-actin (TE8, TE10, TE13, TE14)

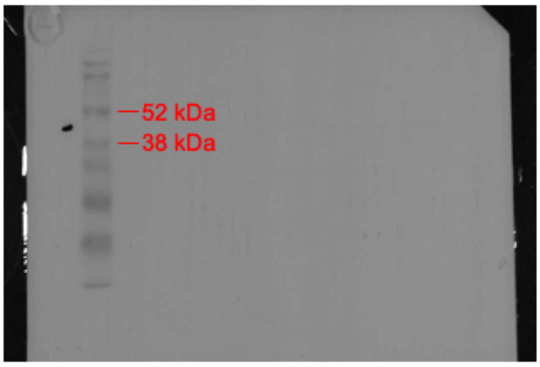

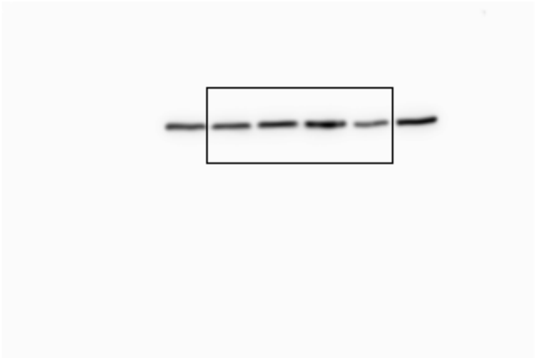

Figure 2B_8-5

B-actin (TE8, TE10, TE13, TE14)

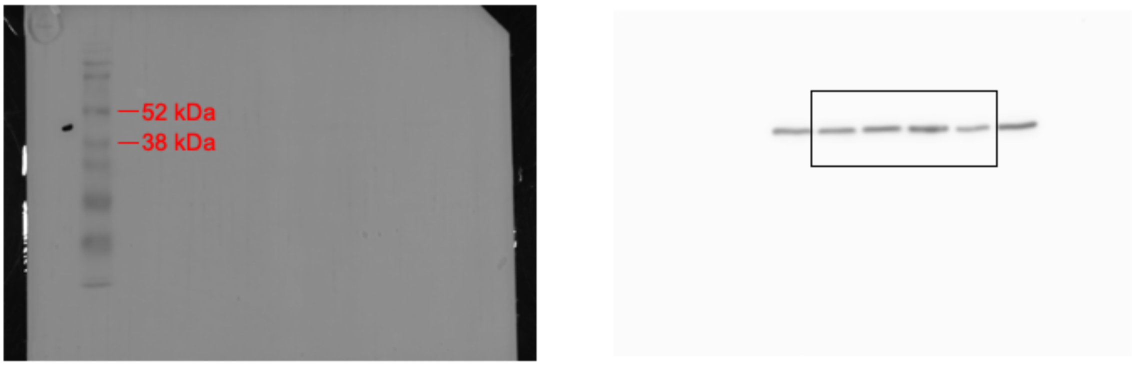

Figure 2B_9-1

HER2 (TE15, OE19, OE33)

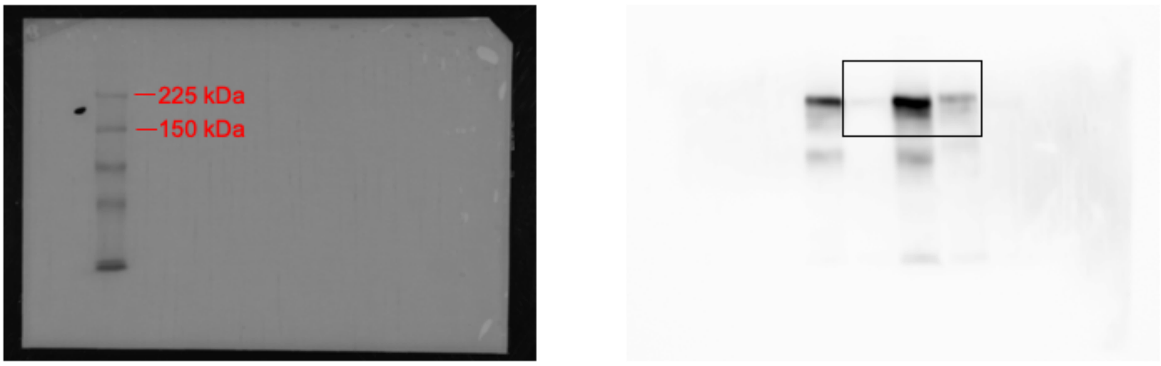

Figure 2B_9-2

HER2 (TE15, OE19, OE33)

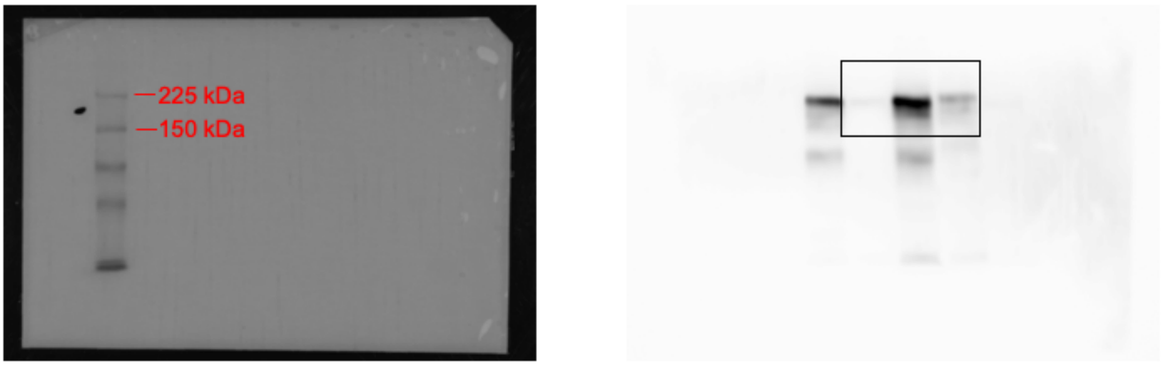

Figure 2B_9-3

HER2 (TE15, OE19, OE33)

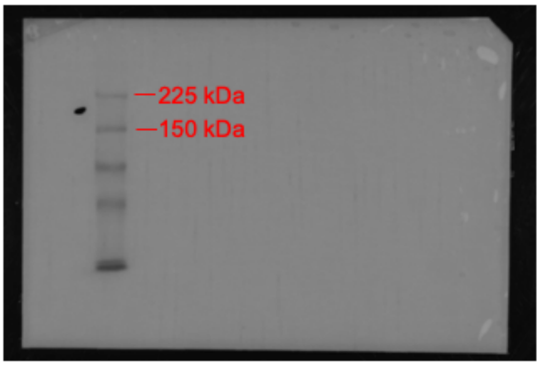

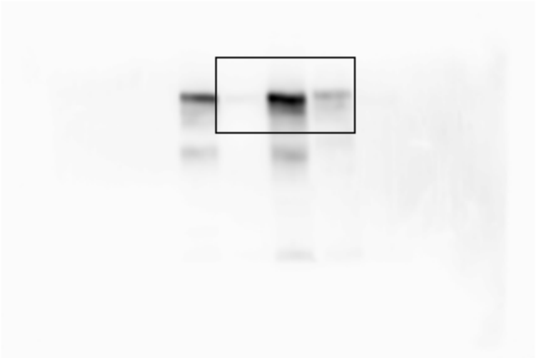

Figure 2B_9-4

HER2 (TE15, OE19, OE33)

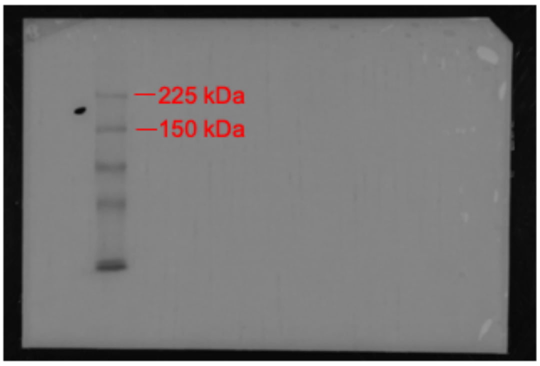

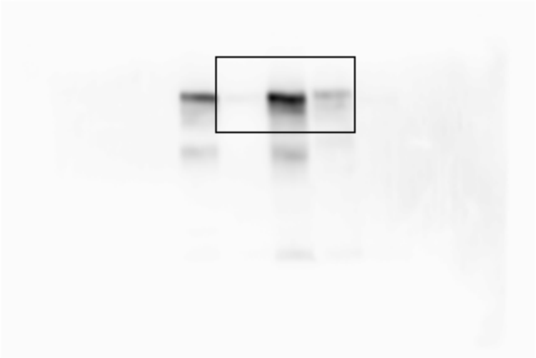

Figure 2B_9-5

HER2 (TE15, OE19, OE33)

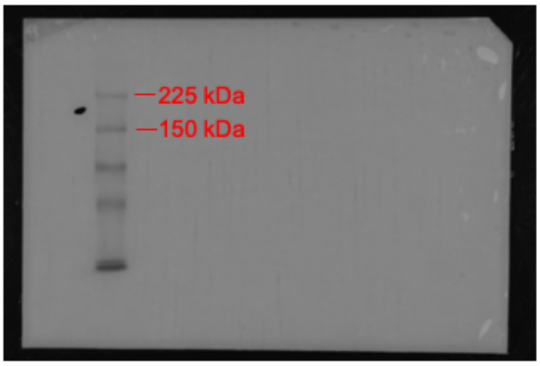

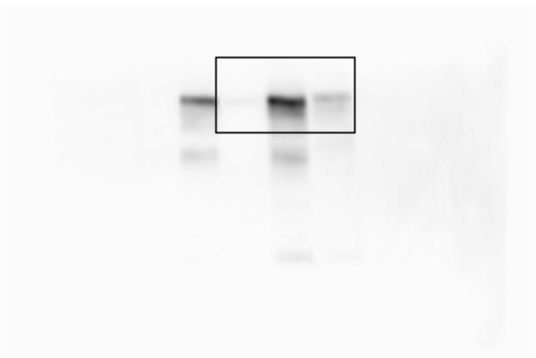

Figure 2B_9-6

HER2 (TE15, OE19, OE33)

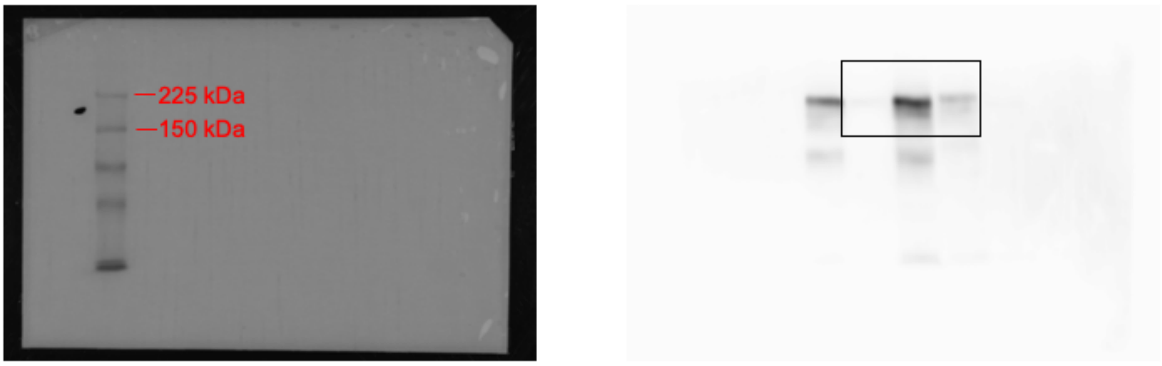

Figure 2B_9-7

HER2 (TE15, OE19, OE33)

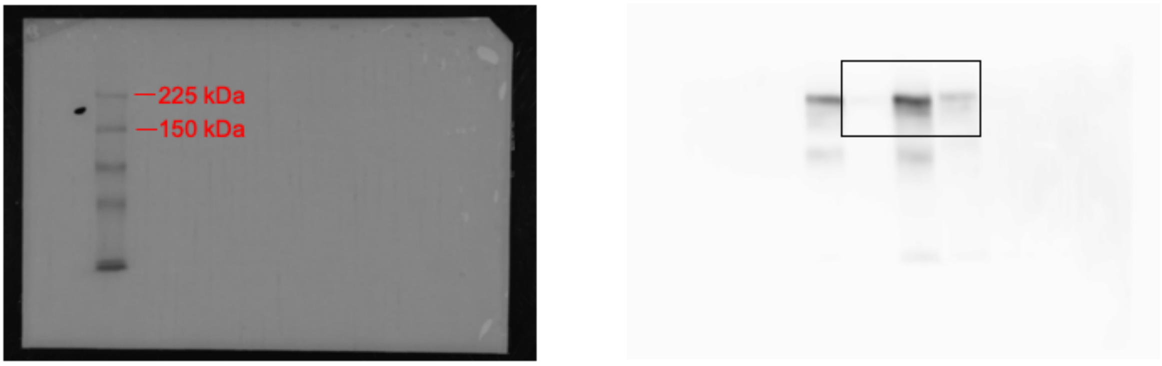

Figure 2B_9-8

HER2 (TE15, OE19, OE33)

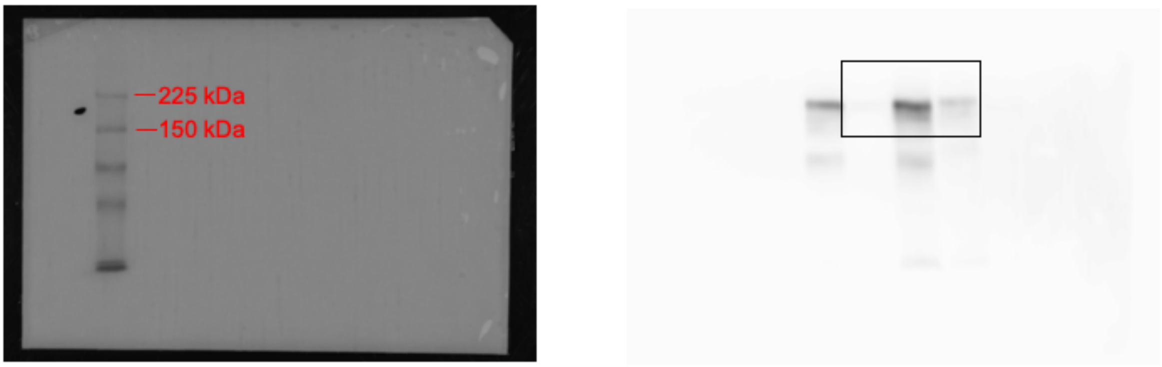

Figure 2B_9-9

HER2 (TE15, OE19, OE33)

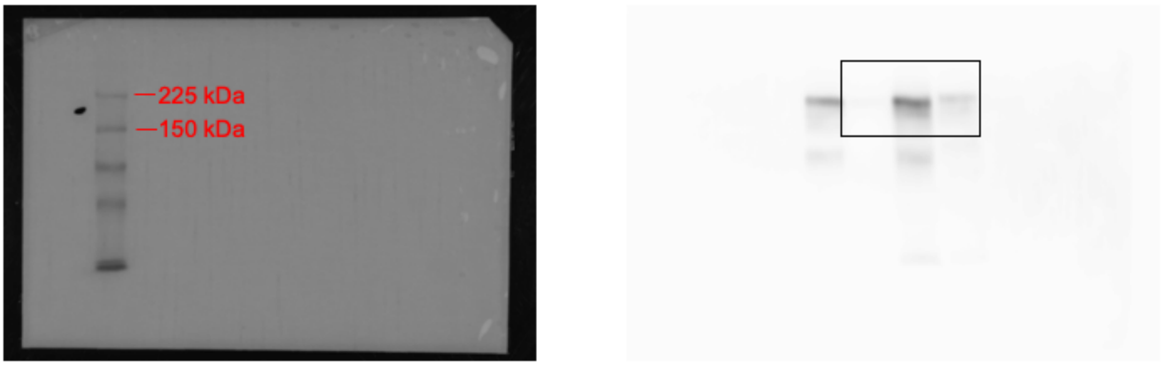

Figure 2B_9-10

HER2 (TE15, OE19, OE33)

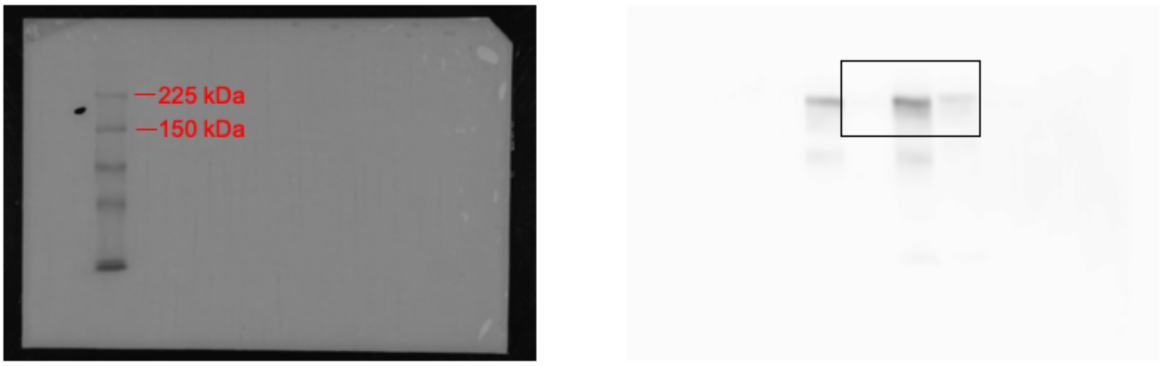

Figure 2B_9-11

HER2 (TE15, OE19, OE33)

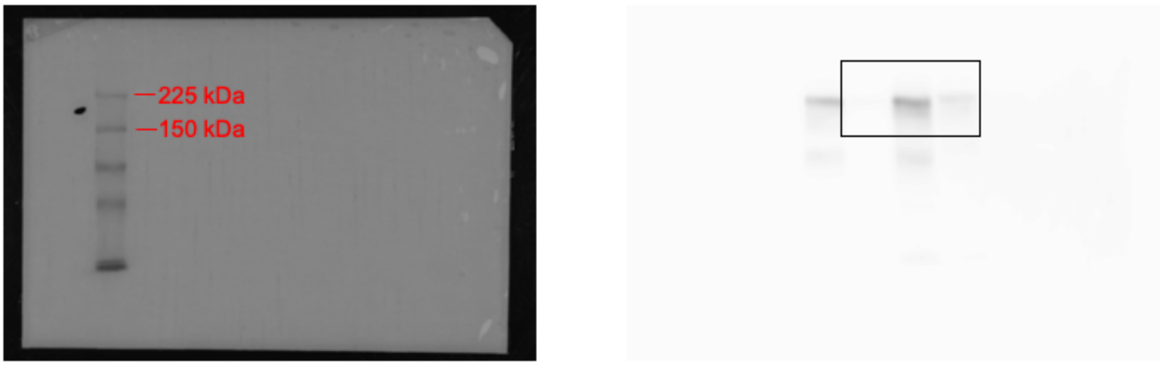

Figure 2B_9-12

HER2 (TE15, OE19, OE33)

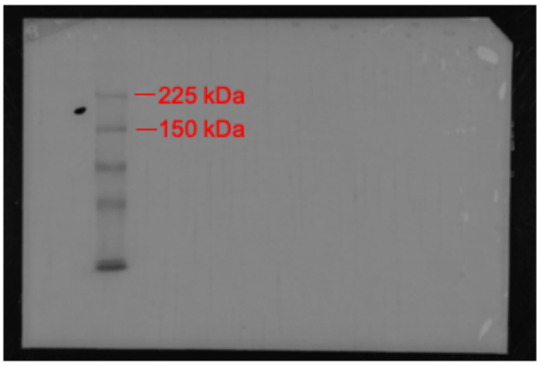

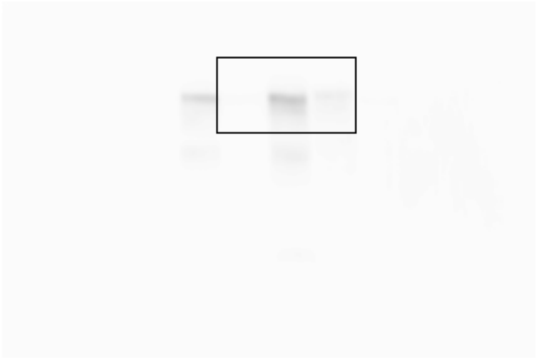

Figure 2B_9-13

HER2 (TE15, OE19, OE33)

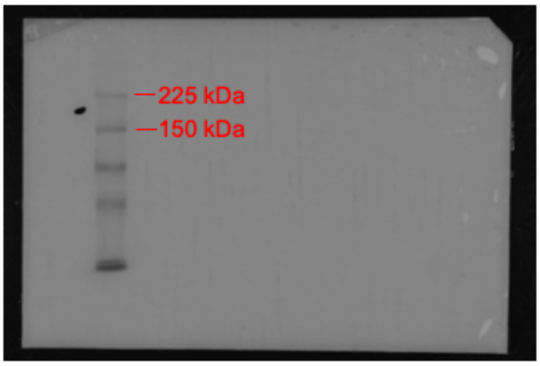

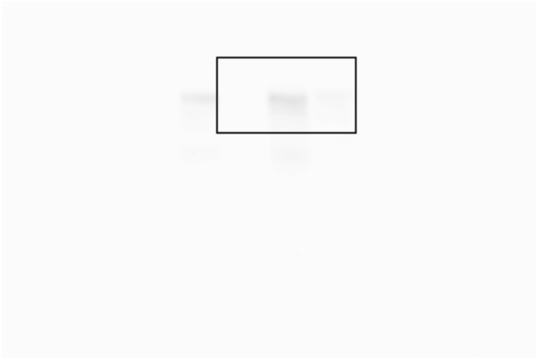

Figure 2B_10-1

B-actin (TE15, OE19, OE33)

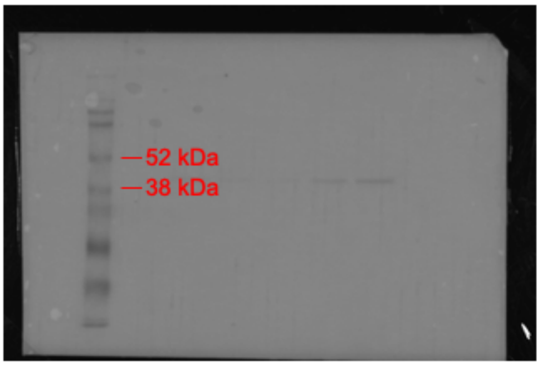

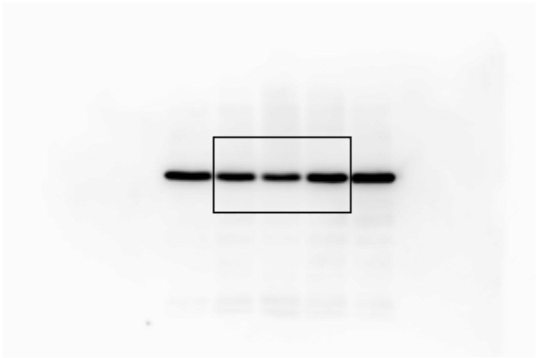

Figure 2B _10-2

B-actin (TE15, OE19, OE33)

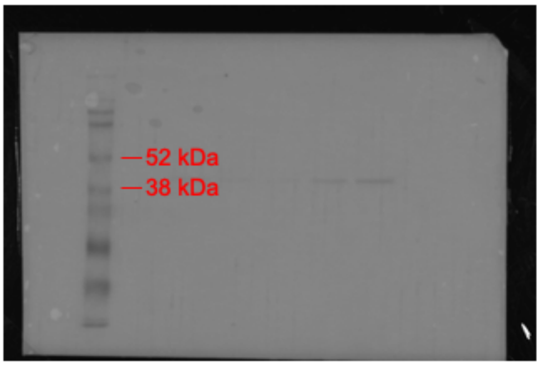

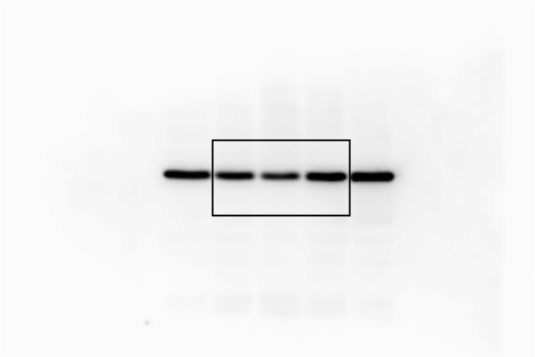

Figure 2B _10-3

B-actin (TE15, OE19, OE33)

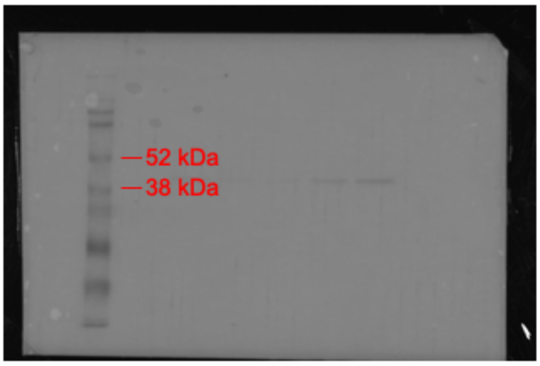

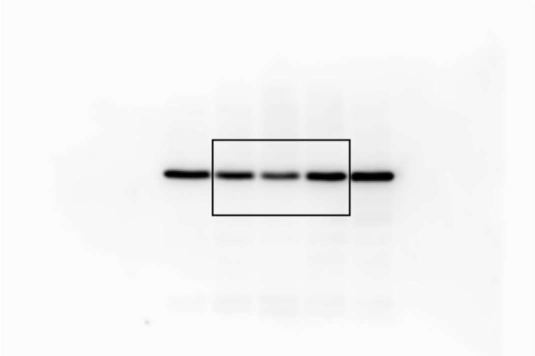

Figure 2B _10-4

B-actin (TE15, OE19, OE33)

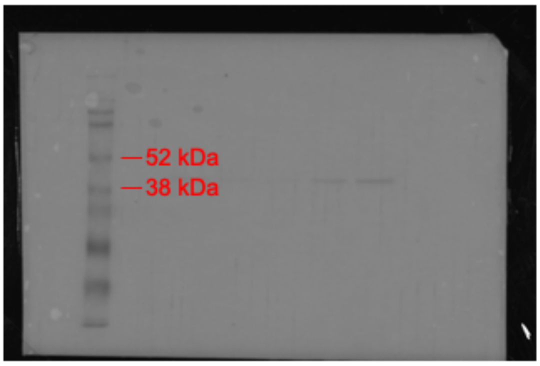

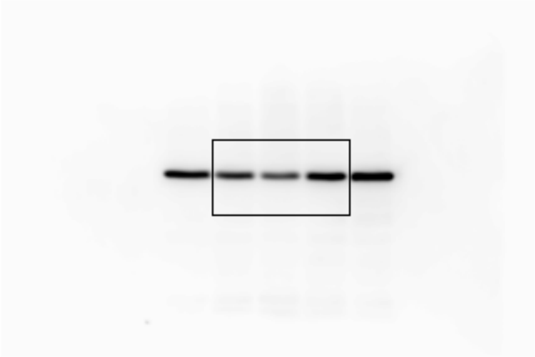

Figure 2B _10-5

B-actin (TE15, OE19, OE33)

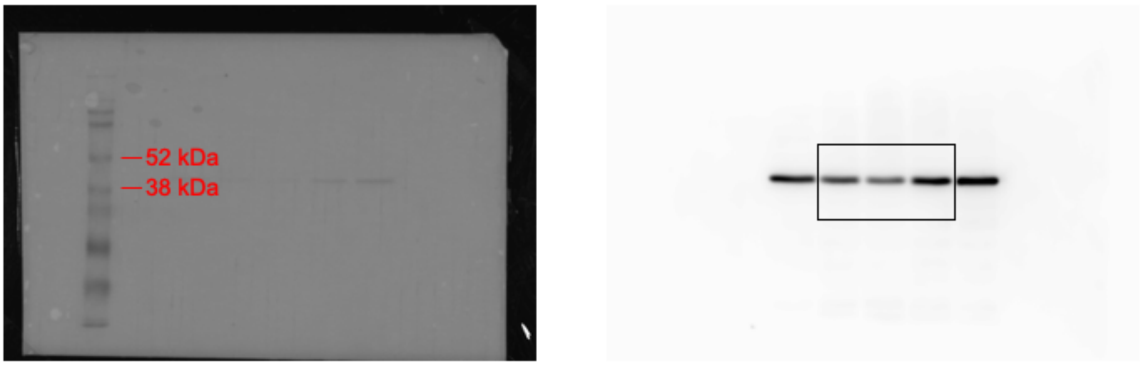

Figure 2B _10-6

B-actin (TE15, OE19, OE33)

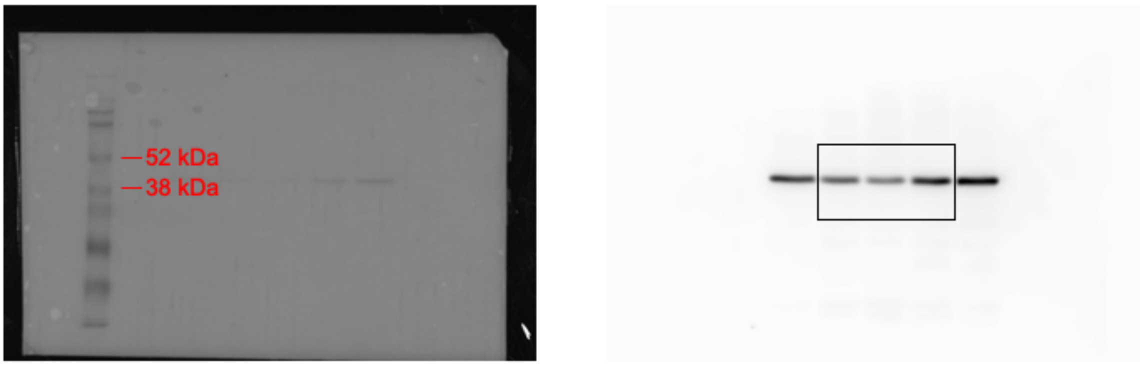

Figure 2B _10-7

B-actin (TE15, OE19, OE33)

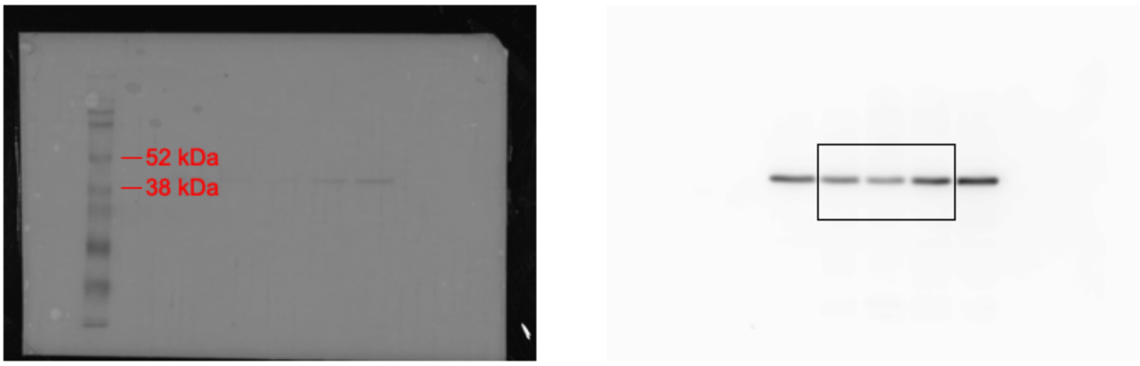

Figure 2B _10-8

B-actin (TE15, OE19, OE33)

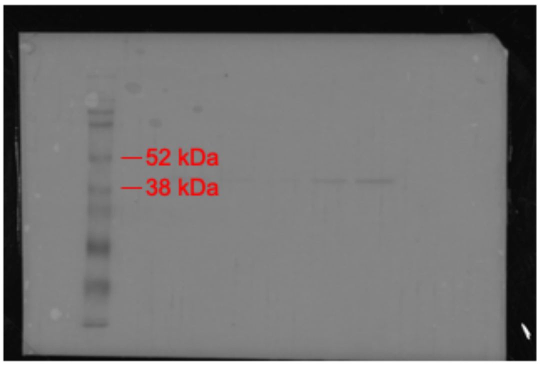

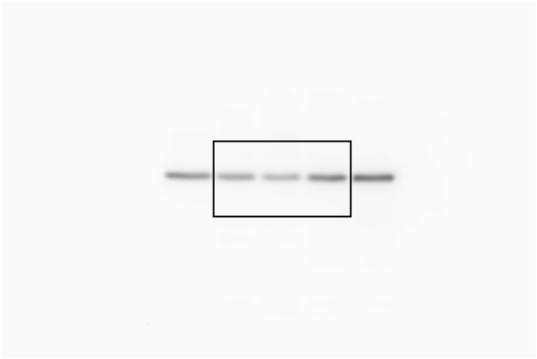

Figure 2B _10-9

B-actin (TE15, OE19, OE33)

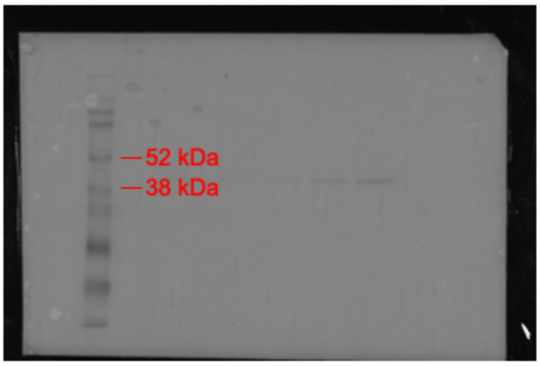

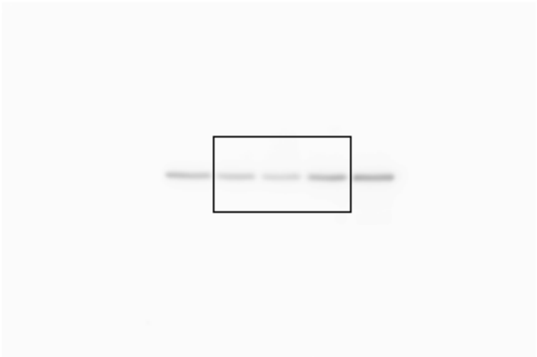

Supplement: Supplementary file 1 — Supplementary Information. [file 41598_2022_24313_MOESM1_ESM.pdf]
